# Quantification of potentially toxic compounds in anhydrous cements: optimization of leaching by thermal analysis for sustainable production of cements

**DOI:** 10.1007/s11356-026-37769-x

**Published:** 2026-05-04

**Authors:** Bruna Souza Rosa, Samile Raiza Carvalho Matos, Luanne Bastos de Britto Barbosa, Heloysa Martins Carvalho Andrade, Ana Paula Kirchheim, Jardel Pereira Gonçalves

**Affiliations:** 1 Polytechnic School, Federal University of Bahia (UFBA), Salvador, Brazil; 2 Center for Territorial Development (CFDT), Federal University of the South of Bahia, Teixeira de Freitas, Brazil; 3 Laboratory of Catalysis and Materials (LABCAT), Department of General and Inorganic Chemistry, Institute of Chemistry, Federal University of Bahia, Salvador, Brazil; 4 Department of Civil Engineering, Federal University of Rio Grande Do Sul (UFRGS), Porto Alegre, Brazil; 5 Polytechnic School, Interdisciplinary Center of Energy and Environment (CIENAM), Federal University of Bahia (UFBA), Salvador, Brazil

**Keywords:** Potentially toxic compounds (PTC), Leaching, Co-processing, Thermal analysis

## Abstract

The co-processing of waste in clinker synthesis is a widely adopted strategy to reduce the consumption of fossil fuels and natural raw materials. However, the incineration of waste may increase the release of potentially toxic compounds (PTCs) and volatile metals, raising concerns for the environment and human health. A major limitation in assessing these risks is the lack of suitable methods to directly quantify PTCs in anhydrous cement; existing approaches are typically limited to hydrated pastes. This study proposes a novel method for quantifying PTC in anhydrous cement by combining leaching tests and thermal analysis. The methodological framework involved: (i) identifying an acid solution capable of sustaining PTC quantification by prolonging reaction kinetics, (ii) selecting the leaching stage, and (iii) defining the optimal solid–liquid contact time. Portland cement samples were characterized, and the reactivity of different solutions was assessed by isothermal calorimetry prior to leaching tests. Results indicated that maintaining a pH between 0.65 and 0.70 optimizes the leaching solution, while the nitric acid/sodium hydroxide ratio must be adjusted explicitly for each cement type. Compared with the standard TCLP method, the proposed approach enabled the detection of 11 metals (Ba, Cr, Cu, Zn, Pb, Ni, La, V, Co, Sb, Mo, and Sn), demonstrating the effectiveness of the refined methodology.

**Supplementary information:**

The online version contains supplementary material available at 10.1007/s11356-026-37769-x.

## Introduction

The production of Portland cement (PC) is a major contributor to global carbon dioxide emissions, releasing approximately 0.44 tons of CO_2_ per ton of cement produced (IEA 2024; SNIC 2019). Without mitigation measures, global CO_2_ emissions are projected to increase 40% by 2050. Strategies such as the co-processing of waste as an alternative raw material and energy source, as well as the partial substitution of cement with supplementary cementitious materials (SCM), have been explored to reduce these impacts (Schneider et al. 2023).

Co-processing waste in clinker synthesis offers clear advantages, including the diversion of waste from landfills and cost reduction. However, this practice also increases the incorporation of potentially toxic compounds (PTC) in cement composition. PTCs are trace metals typically present as oxides, such as Ba, Cr, Cu, Zn, Pb, Ni, La, V, Co, Sb, Mo, and Sn (Stafford et al. 2015; Yan et al. 2010). Their release into the environment raises significant concerns due to risks of air, soil, and water contamination, as well as human health impacts. Owing to their bioaccumulative nature (Belato 2013; Sato 2004), long-term exposure of construction and industrial workers to PTCs can lead to respiratory complications and dermatological diseases (Lv et al. 2018; Van Der Sloot 2002).

Environmental risks assessments of PTCs in cement commonly rely on standardized leaching tests. Among these, the Toxicity Characteristic Leaching Procedure (TCLP), established by the U.S. Environmental Protection Agency (U.S. EPA, 2012), is the most widely adopted. A summary of the main leaching protocols is provided in Table S1 (Online Resource 2). Despite their broad application, these standards present limitations when applied to cementitious systems. They were originally designed for waste leaching (Espinosa and Tenório 2000; Ract et al. 2003; Wang et al. 2010) and therefore do not adequately account for the specific chemical characteristics of cement.

In particular, these methods are generally applied to hydrated pastes, under conditions such as leaching solution type, solid–liquid contact time, and pH ranges (3 < pH < 5) that promote hydration reactions. These reactions compromise the representativeness of results by forming hydrated phases that encapsulate part of the available PTCs, thereby underestimating potential risks (Espinosa and Tenório 2000; Ract et al. 2003; Wang et al. 2010). Consequently, there are currently no standardized procedures for quantifying PTCs in anhydrous cement.

Previous research has predominantly focused on the leaching of hydrated cement (Espinosa and Tenório 2000; Sato 2004; Wang et al. 2010). However, hydration significantly limits detection, since early-formed phases such as calcium silicate hydrated (C-S–H) and ettringite immobilize part of the metals. In contrast, leaching in the early stages of hydration, or directly in anhydrous cement, provides more accurate insights into the mobility of PTCs, as these compounds are more readily available before being incorporated into hydrates.

With respect to the *leaching medium,* two main categories are reported: acidic and alkaline. Acidic solutions, such as hydrochloric, nitric, or sulfuric acid, enhance leaching efficiency because their concentration directly influences the dissolution rate (Navarro et al. 2007; Tsai and Tsai 1998). Nevertheless, several standardized protocols recommend deionized water as the solution to the leaching. This practice promotes rapid formation of hydration product, resulting in near-neutral pH conditions that do not favor metal solubility. Similarly, the extended *solid–liquid contact times* used in current methods (typically > 20 h) overlook the fact that hydration reactions occur within the first hours: C-S–H and ettringite begin forming within approximately2 h, near the end of the induction period (IP), while in situ X-ray Diffraction XRD studies show ettringite formation within minutes (Hewlett and Liska 2004).

The relationship between *pH and metal solubility* further highlights the inadequacy of current protocols. Metal leaching generally increases under acidic conditions (Engelsen et al. 2009, 2010), owing to the tendency of many metals to form soluble oxides in low pH environments (Li et al. 2001; Malviya and Chaudhary 2006). In addition, pH controls the rate, composition, and morphology of hydrated phases (Ramachandran and Grutzeck 1993). While alkaline solutions accelerate cement hydration by favoring calcium ion activity and subsaturation of solid phases, acidic solutions tend to delay hydration (Chen et al. 2018). Ramachandran and Grutzeck (1993) showed that high pH promotes the precipitation of calcium-rich hydrates, whereas acidic conditions reduce hydrate formation. Similarly, Thomas and Double (1981) demonstrated that ethylenediaminetetraacetic acid (EDTA) in acidic media increases silicon release and retards hydration, a behavior explained by the protective membrane theory. Other studies corroborate this effect, reporting extended induction periods under acidic conditions (Ramachandran and Grutzeck 1993; Singh et al. 2003; Wildling et al. 1984).

Various mechanisms have been proposed to explain this retardation. Initially, it was attributed to the formation of a protective barrier, in which calcium removal by acid releases silicate ions, thickening the membrane and delaying hydration (Thomas and Double 1981). However, more recement work emphasizes the geochemical dissolution model (Chen et al. 2018; Juilland et al. 2010, 2017; Nicoleau et al. 2014; Scrivener et al. 2015; Scrivener et al. 2019) which explains the prolongation of the induction period in acidic environments. Nevertheless, this model has limitations under high water-to-cement ratio conditions, where the concentrations of Ca and Si in solution decrease to low levels despite extensive leaching. Huang et al. (2022) suggest that only surface defects control the dissolution rate in such conditions, especially when retarders modify the solution pH. This perspective reinforces the interpretation that prolongation of the induction period results primarily from inhibited nucleation and growth of hydrates, rather than from the formations of a protective layer.

Taken together, these findings highlight that existing leaching methodologies, while suitable for waste, are inadequate for anhydrous cement, since they induce hydration and fail to capture the true availability of PTCs (Espinosa and Tenório 2000; Ract et al. 2003; Wang et al. 2010). Currently, no standardized procedure exists for quantifying PTCs in anhydrous cement (Engelsen et al. 2009; Kiventerä et al. 2019; Lin et al. 2017; Shi and Spence 2004; Vespa et al. 2006).

To address this gap, the present study proposes a novel and efficient methodology for quantification of PTC in anhydrous cement. By enabling a more accurate assessment of the environmental risks (soil, air, water) and occupational health risks associated with the release of PTCs present in the cement matrix, this method also contributes to the advancement of waste management strategies in the cement industry and supports the development of sustainable solutions for reducing CO₂ emissions.

## Experimental

The development of the leaching method for anhydrous cement was divided into 4 stages: i) characterization of the materials, ii) definition of the solution for the leaching tests, iii) definition of the leaching stage, and iv) leaching tests.

The proposed methodology establishes a reproducible procedure for evaluating the leaching behavior of anhydrous cement. By defining optimal solution chemistry and contact time through calorimetry, this approach captures the maximum solubility of PTCs under the most critical conditions. The method therefore addresses a major gap in environmental risk assessment, enabling more accurate quantification of PTCs inherent to cement production, particularly when waste co-processing or SCM incorporation is involved.

### Materials

Ten cement samples, specified according to the Brazilian standard ABNT NBR 16697:^2018^, were evaluated. These cements were divided into two main groups. Group 1 comprised seven materials: six Portland cements with limestone filler, type CP II-F-32 (CP2-A, B, C, D, E, and F), and one high early strength Portland cement, type CP V-ARI (CPV). Group 2 consisted of four oil well cement samples, type Class G (CG-A, B, C, D)**.** CG cement is a commercial cement, while the others are produced in a laboratory.

The leaching solutions evaluated included alkaline solution (NaOH, 1 M), acidic solutions (HNO₃ at 65% and 20%, and H₂SO₄), and isopropyl alcohol (60%).

#### Characterization of anhydrous cement

The characterization of the cement composition as oxides was performed using the X-ray fluorescence (XRF) method with Bruker's Model S8 Tiger equipment. The chemical composition obtained by the XRF test was corrected by the fire loss index (FP) according to NM18 (2012). Cement mainly comprises CaO, SiO_2_, and Fe_2_O_3_, with these three oxides accounting for 71–76% of the total mass. For GC cement, this sum reaches 84–89.87%. For the minority elements TiO_2_, ZnO, BaO, MnO, V_2_O_5_, Cr_2_O_3_, and PbO, the levels ranged from 1.61% to 2.68%. These potentially contaminating elements come from the co-processing process in the kiln or are associated with the mineral additives used during cement manufacture. In group G cement, SrO, MnO, V_2_O_5_, Cr_2_O_3_, and NiO oxides are present in concentrations ranging from 0.48% to 0.78%.

Cement samples (10.0–10.5 mg) were tested in a Mettler Toledo TGA/DSC 2 thermogravimetric analyzer using an open alumina crucible under flowing nitrogen atmosphere at a heating rate of 20 °C min − 1 from 40 to 1000 °C. Prior to heating, an isothermal step was maintained at 40 °C for 10 min to remove physically adsorbed moisture. The instrument was calibrated for temperature and mass according to the manufacturer’s procedure. Figure S1 and Figure S2 (Online Resource 1) illustrate the thermal decomposition of the phases present. The temperature ranges and quantified values for gypsum, Portlandite (CH), and limestone of all the cements analyzed are summarized in Online Resource 2 (Table S3 and Table S4). The quantification of the gypsum content is determined using the ratio between the decomposition of gypsum and the corresponding stoichiometric mass of CaSO_4_·2H_2_O, as described by Dweck et al. (2016). Calcium hydroxide was quantified according to its stoichiometric decomposition equation and the molar masses of the substances. Group 1 cements were slightly hydrated with portlandite contents ranging from 0.45—2.34% CH due to the hydration of free lime (CaO), which is hydrophilic and forms calcium hydroxide (Ca(OH)_2_ (s) → CaO(s) + H_2_O(l)). CP2-C had the highest CH content, while CG cemented the lowest. For CG cement, the amount of CH is considerably irrelevant, with levels ranging between 0.002% and 0.004%. Regarding calcium carbonate (CaCO_3_), the commercial cement had limestone filler contents of up to 19–24% for CPII-F and 7.94% for CPV. The highest carbonate values are observed in CPII-F cement, which has a filler content of up to 25%. For the G cement, the levels ranged from 0.39% to 0.48%.

The chemical characterization (in oxides) and mineralogical composition of the cement samples are shown in Table 1.

**Table 1:** Characterization of cement

Normalized oxide composition											
**Oxide composition (%)**	CP2-A	CP2-B	CP2-C	CP2-D	CP2-E	CP2-F	CP V	CG-A	CG-B	CG-C	CG-D
CaO	57,75	57,55	60,67	54,46	60,14	62,42	58,59	63,93	65,12	64,51	65,34
SiO_2_	12,43	15,07	13,74	14,76	11,67	13,97	14,99	19,09	19,04	18,91	18,53
SO_3_	3,59	4,55	3,04	3,98	3,81	3,43	4,81	2,94	2,76	2,07	1,95
MgO	4,56	3,41	2,07	2,3	3,96	1,34	3,12	1,17	0,32	0,34	0
Al_2_O_3_	3,06	3,1	3,38	3,08	3	2,96	2,89	3,37	3,73	3,84	2,93
Fe_2_O_3_	1,68	2,68	2,07	2,2	1,67	2,66	2,67	3,86	5,71	4,84	4,88
K_2_O	0,96	1,77	1,14	0,74	1,14	0,78	1,73	0,38	0,02	0,01	-
SrO	0,04	0,18	0,05	0,12	0,04	0,17	0,19	0,16	0,26	0,27	-
TiO_2_	0,21	0,18	0,24	0,22	0,21	0,21	0,16	0,23	0,02	0	-
P_2_O_5_	0,28	0,13	0,48	0,11	0,27	0,05	0,13	0,09	0,11	0,09	-
Na_2_O	0,25	0,06	0,16	0,31	0,29	0,27	0,1	0,29	0,06	0,03	-
ZnO	58 ppm	0,05	0,03	0,03	65 ppm	0,01	0,05	0,04	66 ppm	46 ppm	64,36 ppm
BaO	0,02	0,04	-	0,03	-	-	0,04	-	-	-	-
MnO	0,02	0,04	0,02	0,22	0,02	0,06	0,04	0,06	0,02	0,01	-
Cl	0,02	0,04	0,04	0,02	0,02	0,02	0,04	0,02	-	0,01	-
CuO	-	0,03	0,02	0,03	22 ppm	32 ppm	0,03	72 ppm	34 ppm	31 ppm	-
V_2_O_5_	-	0,03	-	-	-	0,01	0,03	0	0,06	-	-
ZrO_2_	0,01	0,01	0,01	0,02	0,01	0,02	0,01	0,01	83 ppm	-	-
Cr_2_O_3_	0,01	72 ppm	0,02	0,01	0,01	0,01	81 ppm	0,02	92 ppm	94 ppm	-
NiO	57 ppm	59 ppm	81 ppm	55 ppm	65 ppm	41 ppm	70 ppm	64 ppm	0,05	-	-
MoO_3_	-	63 ppm	-	0,01	-	-	69 ppm	-	-	-	-
Ag	-	46 ppm	45 ppm	40 ppm	63 ppm	55 ppm	48 ppm	-	65 ppm	-	49,73 ppm
PbO	-	36 ppm	-	30 ppm	-	-	31 ppm	-	-	-	-
Rb_2_O	20 ppm	25 ppm	33 ppm	12 ppm	27 ppm	22 ppm	22 ppm	-	-	-	-
As_2_O_3_	-	16 ppm	-	23 ppm	-	-	19 ppm	-	-	-	-
CeO_2_	-	0,02	-	-	0,03	-	-	-	-	-	-
CoO	-	-	-	-	-	-	-	-	-	-	-
La_2_O_3_	-		-	-	-	-	-	4,33	0,29	-	0

### Development of the new leaching method

The proposed leaching method was developed through successive experimental stages. Initially, the solution to be used in the leaching tests was defined, considering the nature of the leaching agent, pH, and metal solubility. This stage consisted of determining the most appropriate extraction solution, since previous studies indicate that the leaching of certain elements is directly related to the pH of the material (Engelsen et al. 2009; 2010). For this purpose, pH evolution was evaluated.

In the following stage, the leaching stage and the contact time were established. The optimal leaching stage and contact time were investigated based on calorimetry tests. The objective was to investigate the greatest potential for element detection, to avoid the retention of PTCs in the pore solution. To this end, calorimetry tests were carried out using solutions suggested by the standard with 3 < pH < 5 and optimized solutions.

**Fig. 1:**
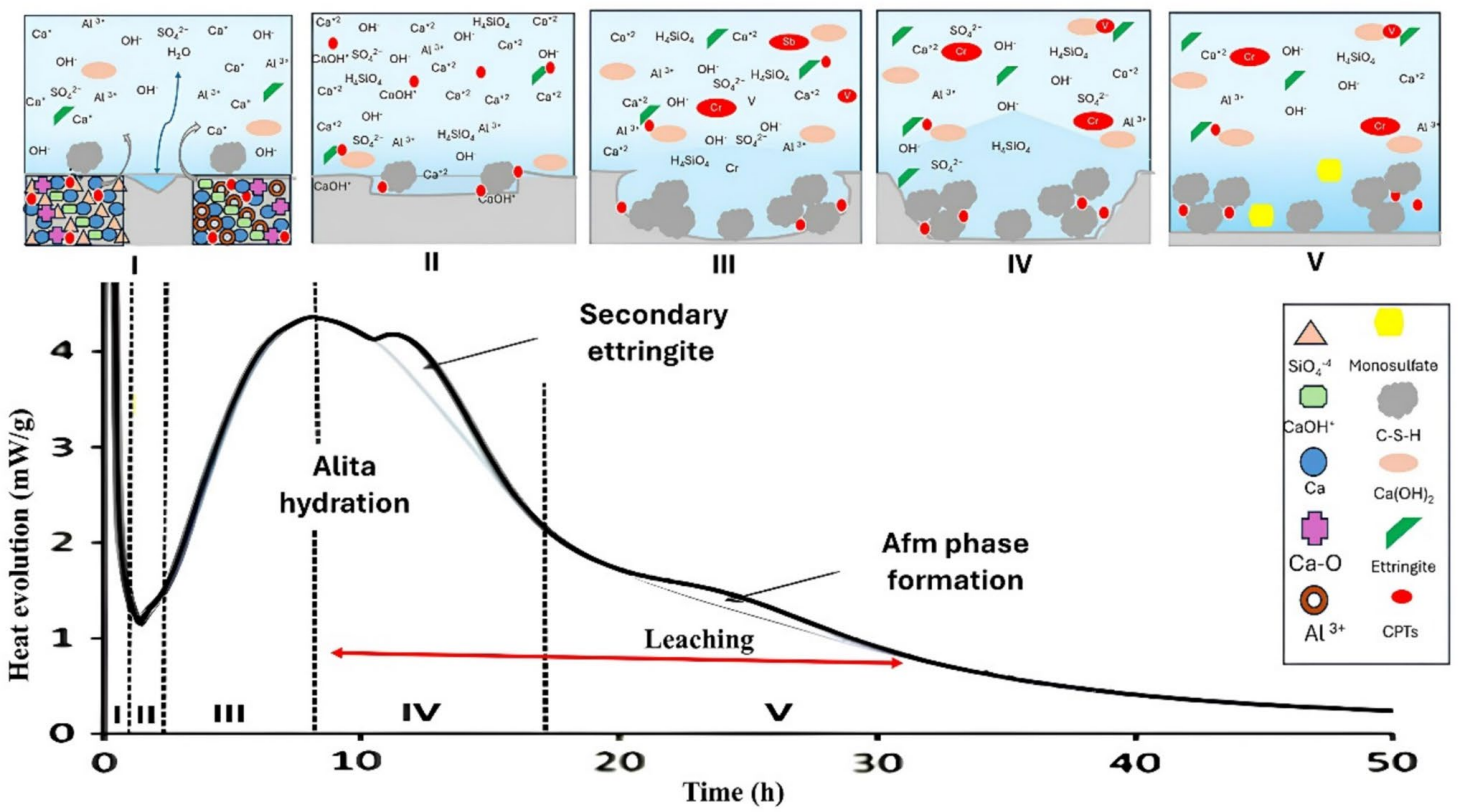
Hydration kinetics with deionized water or 3 < pH < 5

Figure 1 shows the hydration evolution of anhydrous cement in contact with solutions with a pH range of 3–5, in line with experimental procedures reported in the literature. Initially (Fig. 1), during the first few minutes (stages I and II), rapid dissolution of highly reactive phases occurs, particularly C₃A, which reacts with calcium sulfate in solution to form an amorphous aluminate gel and nucleate ettringite at the grain boundaries. This process is reflected in the initial heat peaks.

**Fig. 2:**
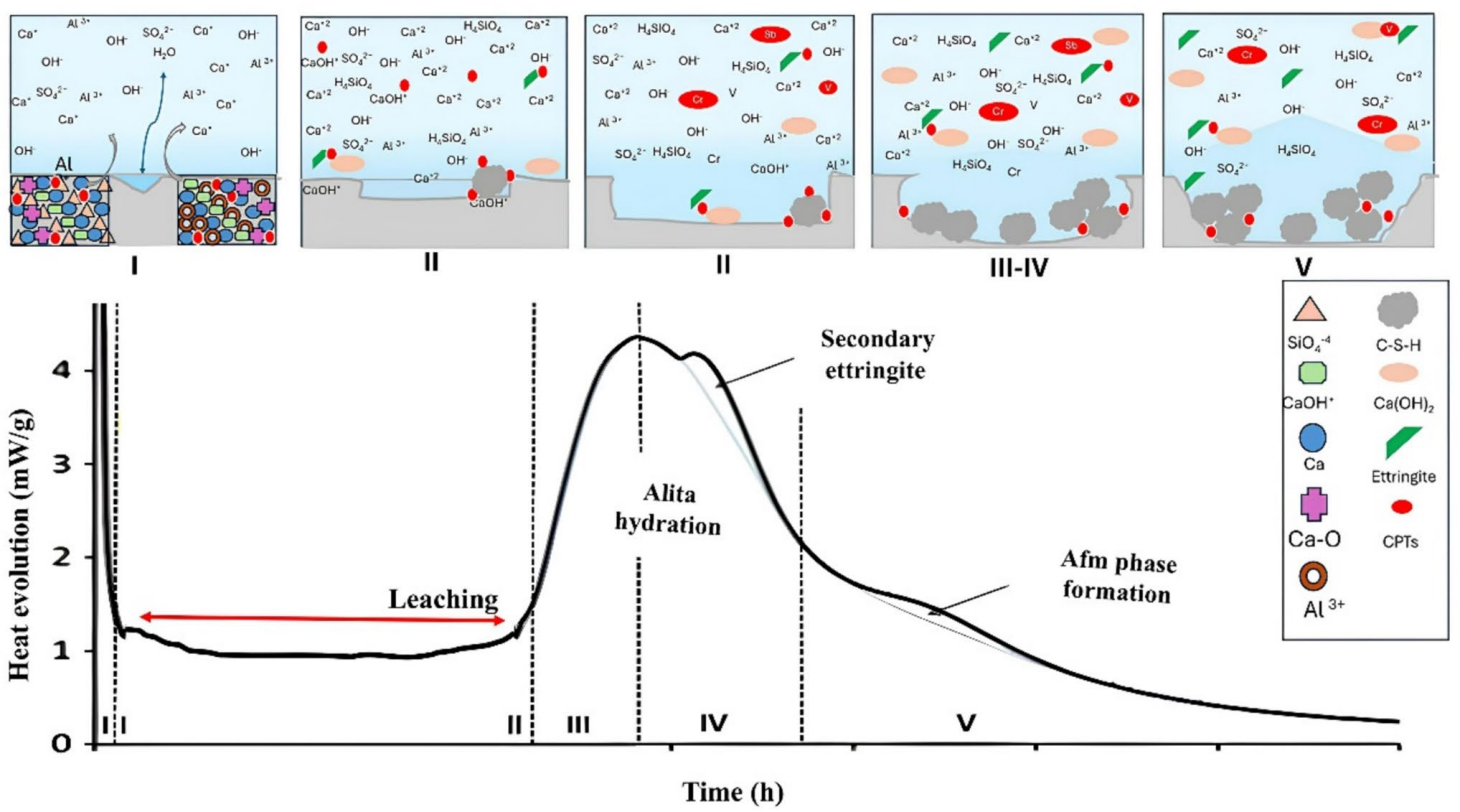
Extending hydration kinetics with an optimized solution

During the induction period (III), a significant reduction in the heat release rate is observed, corresponding to the formation of an initial product layer that slows diffusion and temporarily inhibits further reactions (plateau effect). The subsequent acceleration period (IV), lasting up to ~ 10 h, is marked by the intense hydration of C₃S, accompanied by the formation of external C-S–H bonds and the release of the main heat peak. From this point onward, in the deceleration period (V), C₃A hydration continues with the formation of secondary ettringite and its conversion into AFm-type phases (e.g., monosulfate). In contrast, C₃S continues to hydrate internally, progressively filling pores with fibrous C-S–H. These processes are associated with a milder but sustained heat release that can persist for days (Quennoz and Scrivener 2013; Scrivener et al. 2015; Scrivener et al. 2016). As shown in Fig. 1, the hydration of anhydrous cement evolves progressively under experimental conditions with solutions in the range 3 < pH < 5. Conversely, Fig. 2, presents the hypothesis that the optimized solution can extend the induction period, during which leaching is most active, thus maintaining the potentially toxic compounds (PTCs) in the pore solution for improved quantification. Thus, the main distinction between the proposed methodology and those established in the standards applicable to wastes concerns the hydration stage at which the leaching test is performed. While the standard methods prescribe carrying out this procedure at hydration stage IV, the proposed methodology establishes its execution at stage II.

Finally, the leaching tests were carried out based on the parameters defined in the previous stages, namely: the nature of the leaching agent, pH, leaching stage, and contact time. The methodological procedure adopted is described below.

#### Definition of the solution for the leaching tests: Nature of the leaching, ph, and metal solubility

Nine solutions were prepared using sodium hydroxide (NaOH), nitric acid (HNO_3_ 65%, HNO_3_ 20%), sulfuric acid (H_2_SO_4_), and isopropanol alcohol. Based on the criteria of leachate composition, pH, and metal solubility, three preliminary tests (Tests 1, 2, and 3) were conducted to assess pH variation. The proportions of the reagents in tests 1 and their pH are shown in Table 2, and the proportions of the reagents in solutions for tests 2 and 3 are shown in Table S9. These tests involved adjusting the proportions of nitric acid, sulfuric acid, and isopropanol to achieve different pH values in CPV cement. In addition, isothermal calorimetry was performed to evaluate the effects of these variations on the hydration behavior of anhydrous cement. The experimental steps were as follows:

i. Cement samples were previously dried at 38 °C and conditioned.
ii. Solutions were prepared with specific amounts of acid and base (Table 2)
iii. CPV cement was mixed with the prepared solutions at a solid–liquid ratio (S/L) of 1:10. Specifically, 4 g of anhydrous cement was added to 40 mL of solution. That is, each solution was prepared by adding the amount of reagent from the table (Table 2, Sect. 3.1) to 40 mL of deionized water.
iv. The mixture was initially stirred at 14,000 revolutions per minute (rpm) for 1 min, followed by a second stage of stirring at 21,000 rpm for another minute, totaling 2 min of mixing.
v. The pH of the mixture was measured at two points: (i) immediately after mixing (2 min) and (ii) after an additional 4–5 min of contact with cement to monitor pH evolution. Out of the nine prepared solutions, seven were selected for calorimetric analysis (Sect. 3.1).
vi. After measuring the pH, 800 ppm of 50% HNO₃ was added to Solutions 5–9 so that each sample would have a 2% acidity. This was done to limit the rapid increase in pH caused by the dissolution of ionic species (K⁺, Na⁺) and the release of Ca^2^⁺ and SO₄^2^⁻ from CaSO₄.
vii. Subsequently, approximately 6–7 g of the cement–solution mixture was placed in ampoules and inserted into the calorimeter.

**Table 2:** Optimization of solutions for leaching tests—Test 1

Tests 1							
Solution	Type of cement	1M NaOH (g)	HNO_3_ 65% (mL)	HNO_3_ 20%	H_2_SO_4_ (mL)	Isopropanol (mL)	pH of the solution
1	CPV-ARI	0,8g	1,375mL	-	-	-	5,72
2	CPV- ARI	0,2g	1,375mL	-	-	-	0,68
3	CPV- ARI	0,4g	1,375mL	-	-	-	0,89
4	CPV- ARI	0,4g	1,375mL	-	-	15mL	12,77
5	CPV- ARI	-	-	1,375mL	1,0mL	-	0,88
6	CPV- ARI	-	-	1,0mL	1,0mL	-	0,73
7	CPV- ARI	-	-	1,0mL	-	10,0mL	1,12
8	CPV- ARI	-	-	2 drops	2 drops	-	1,54
9	CPV- ARI	-	-	2,0mL	-	-	1,55

#### Definition of the leaching stage and contact time

Isothermal calorimetry was conducted to evaluate the hydration kinetics of anhydrous cement in contact with various leaching solutions (deionized water, NaOH, HNO_3,_ H_2_SO_4_, and isopropanol). This study aimed to determine the most suitable hydration stage for the leaching test and establish the optimal contact time. A TA Instruments TAM Air calorimeter was used, operating at 23 °C for 72 h. The heat flux (mW/cement) and the accumulated heat (mW/cement) were measured by J/g_cement_.

Two main criteria guided the definition of the leaching stage: (i) prolonged hydration kinetics and (ii) a lower rate of hydrated phase formation (Fig. 1 and Fig. 2). Contact time was considered a critical parameter, as it directly influences the effectiveness of PTC detection by enhancing mass transfer between the solid matrix and the leaching solution. The procedure involved the following steps:

i. Using the cement and deionized water mixture from the previous step (2.2.1), approximately 6–7 g of the cement–solution mixture was placed in ampoules and inserted into the calorimeter.
ii. Calorimetric curves were analyzed and compared to those obtained using deionized water and the optimized solution (Fig. 1 and Fig. 2). From these curves, the induction period (LIP) and the initial and final setting times were determined. The latter values were obtained from the derivative of the heat flow curve, where the maximum value corresponded to the end of setting and zero corresponded to the beginning (Hu et al. 2014).
iii. The leaching stage was defined as the induction period, characterized by the plateau effect associated with limited formation of new hydration phases (Fig. 2).

#### Leaching tests

Two types of leaching tests were conducted at this stage: (i) tests based on standard protocols (NBR 10005 and TCLP 1311) and (ii) optimized tests using the solution defined in Sect. 2.1.2.

The first leaching test followed the NBR 10005 (ABNT 2004) and TCLP 1311 (USEPA 1992) procedures. The leachate pH was adjusted with small amounts of 50% (v/v) HNO₃ to reach pH 2 (Table S5) prior to ICP-OES analysis (9800 Series, Shimadzu). Metals selected for analysis were consistent with those listed in ABNT NBR 10004:2004 (ABNT 2004), Resolution No. 396 (Brasil 2008) and other relevant standards (Hewlett and Liska 2004).

For the optimized leaching test, the solution with the longest induction period, identified in Sect. 2.1.2, was selected. To prepare the tests, 6 g of cement was mixed with 60 mL of extractant solution in a 250 mL glass beaker (solid-to-liquid ratio of 1:10). Three experimental conditions were tested: TL.1, TL.2, and TL.3. The difference between the tests lies in the agitation procedure. According to the literature, agitation plays a key role in leaching tests, as it is the primary factor driving mass transfer between the sample and the leachate. Agitation helps accelerate the dissolution of elements or compounds in the solid material and allows chemical equilibrium to be reached more quickly.

- **TL.1**: manual mixing of cement and leaching solution in a beaker using circular movements for one minute.
- **TL.2 and TL.3**: mechanical stirring at 14,000 rpm for 1 min, followed by 21,000 rpm for an additional minute (total mixing time: 2 min).

The post-mixing pH of all solutions was measured (Tables S7 and S8, Online Resource 2). Only sample TL.2 was acidified with 50% HNO₃, added at a concentration of 2% (v/v) of the final sample volume, which corresponds to a final nitric acid concentration of 800 ppm. After mixing, all suspensions were homogenized using a mechanical mixer (Marconi, MA 420) at 230 ± 2 rpm and maintained at 25 ± 2 °C. The contact time was defined based on the duration of the induction period (LIP).

The optimized leaching was carried out during the stage characterized by the lowest rate of hydrate formation, i.e., the induction period. The contact time of 20 h was defined according to the LIP duration observed for leaching solution 2 (pH 0.68; reagents: 1 M NaOH + 65% HNO₃). Finally, the leachate pH of each sample was measured before analysis by ICP-OES, using a 9800 Series ICP-OES spectrometer (Shimadzu), equipped with a Seaspray nebulizer and cyclonic spray chamber, both operated with high-purity argon (> 99.996%, White Martins, São Paulo, Brazil).

#### Proposal for a method to quantify PTC through leaching tests

Based on the experimental procedures and results obtained, a methodological proposal was established to optimize the quantification of Potentially Toxic Compounds (PTCs) in anhydrous cement using leaching tests (Sect. 3.5).

## Results and discussions

### Defining the solutions for the leaching solution

The solutions were chosen based on the limiting factors for leaching, including the nature of the leaching solution (acidic or alkaline), the solubility of the metals, and the pH. The following leaching solutions were selected (Table 2): i. Nitric acid (HNO_3_) and sulfuric acid (H_2_SO_4_) solutions, due to their compatibility with Potentially Toxic Compounds (PTC), as set out in the Shimadzu ICP-OES—ASC-9800 equipment manual (Table S6- Online resource 2); ii. NaOH solutions are more effective at leaching V and Al, while NH_3_ and Na_2_CO_3_ are particularly effective at leaching Ni, as demonstrated by Navarro et al. (2007) and Tsai and Tsai (1998). However, NaOH solutions were selected for this study; iii. Isopropanol, because hydration with a solution containing a proportion of isopropanol slows down the hydration kinetics, which will have an impact on the dissolution rate of C_3_S and the growth of C-S–H (Scrivener et al. 2015).

The solubility of metals is strongly influenced by pH. Engelsen (Engelsen et al. 2009, 2010) reported that cationic species (Cd, Cu, Ni, Mn, Pb, Zn) are stabilized at pH > 9, oxyanions (Cr, Mo, Ni) at 4 < pH < 12, and amphoteric species below pH 7. Consequently, cations, anions, and soluble salts exhibit distinct leaching behaviors due to differences in chemical speciation, with their mobilities varying according to the solution pH (Van Der Sloot 2002). Leaching of metal cations is favored under acidic solutions (Figure S3- Online resource1—(US EPA 2012)). which justifies the selection of solutions 1**, 2, 3, 5, 7, 8, and 9**.

#### Optimizing the solution for the leaching tests

Isothermal calorimetry was conducted to identify the optimum hydration stage of the anhydrous cement in contact with each leaching solution, simulating the conditions and guiding the selection of an optimized leaching medium.

**Fig. 3:**
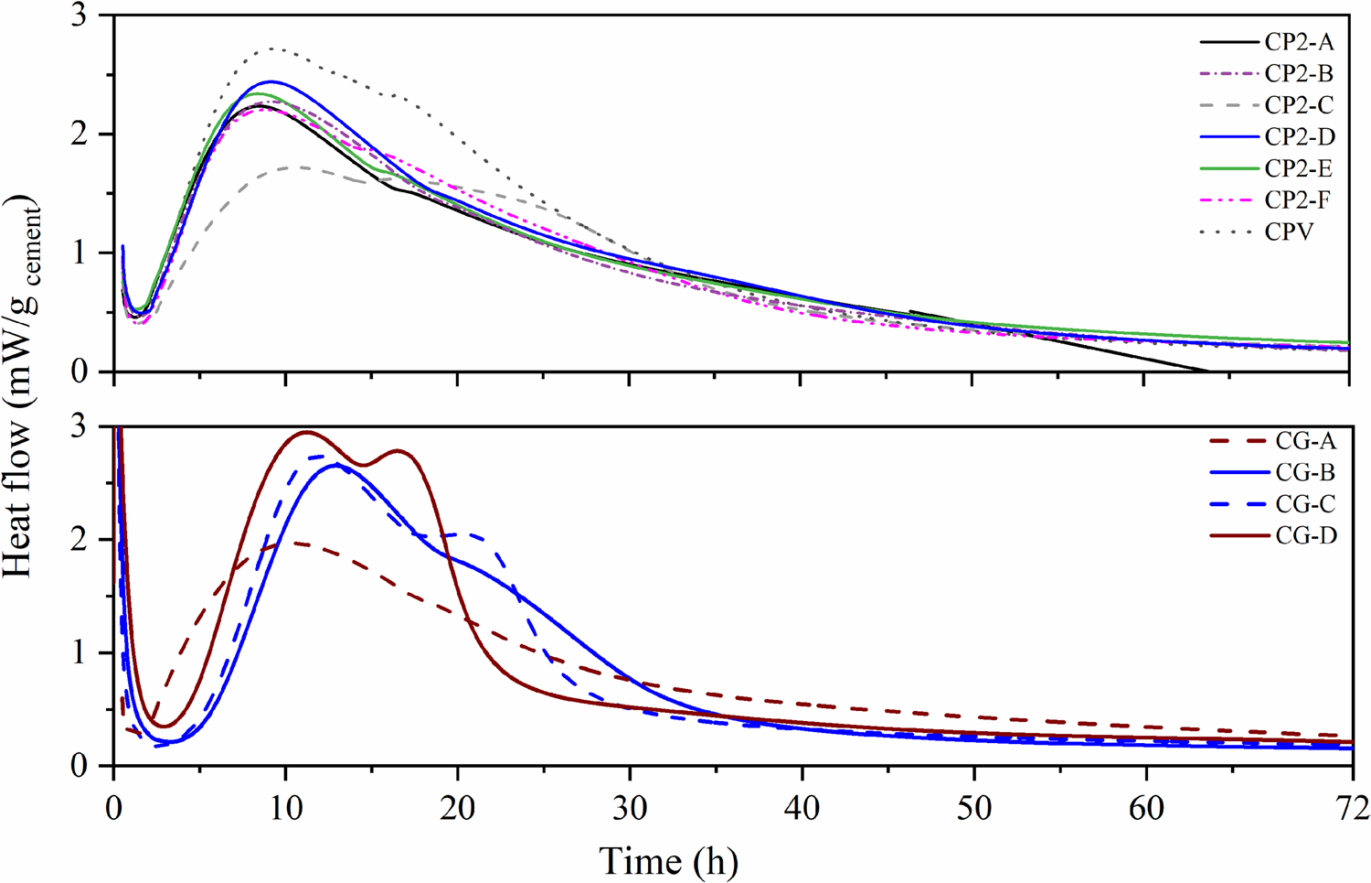
Calorimetry of Group 1 cements with Milli-Q water

Figure 3 shows that the heat flow curves of commercial cements prepared with Milli-Q water exhibited comparable hydration behaviors. The first exothermic peak, associated with silicate hydration, occurred at 10–12 h for all cements. The second peak, linked to aluminate hydration, presented more pronounced shoulders in CP2-C, CP2-F, CP2-E, CP2-A, and CPV, reflecting the resumed hydration of C₃A after sulfate depletion. These results indicate incomplete early C₃A reactions, followed by secondary ettringite formation. Key hydration events for Groups 1 and 2 are summarized in Table 3.

**Table 3:** Hydration kinetics parameters of cement with Milli-Q water solution

Parameters	CP2-A	CP2-B	CP2-C	CP2-D	CP2-E	CP2-F	CPV	CG-A	CG-B	CG-C	CG-D
Initial setting (h)	3,4	3,56	4,56	3,46	3,25	3,51	3,41	4,81	8,84	8,36	7,76
Final setting (h)	8,44	9,25	10,85	9,25	8,44	8,75	9,25	10,45	12,52	11,97	11,21
Duration (h)	5,04	5,69	6,29	5,79	5,18	5,24	5,84	5,64	3,68	3,61	3,45
Duration of induction period- LIP (h)	1,2	1,69	1,2	1,8	1,2	1,51	1,6	1,11	4,28	4,25	2,83
Maximum heat flow (mW/g cement)	2,23	2,27	1,71	2,44	2,33	2,21	2,71	2	2,65	2,74	2,95

According to Table 3, commercial cements showed initial setting times of 3–5 h, final setting times of 5–6.3 h, and induction periods (LIP) of 1–2 h. For Type G cements, the setting time ranged from 4 to 9 h, with durations of 3.63 to 5.64 h and LIP values of 1.11 to 4.43 h. These findings confirm that when cement is dissolved in water, hydrated phases typically form within 20–40 h, consistent with leaching standards. However, this also highlights the need for solutions capable of delaying hydration and stabilizing the induction period, allowing leaching tests to capture PTC release before significant hydrate formation occurs.

#### Definition of the leaching solution: Optimized solutions—tests 1–3

Table 4 shows the results of Test 1, which utilized CPV cement, conducted to assess the impact of reagent contents on pH and calorimetry curves using thermal analysis.

**Table 4:** Optimized solution: Test 1 solution for calorimetry analysis

Solution	pH of the solution	pH after mixing (cement* + solution)	pH after 4 ~ 5 min
1	5,72	12,14	12,2
2	0,68	11,7	11,76
3	0,89	11,79	11,86
4	12,77	-	-
5	0,88	9,51	10,1
6	0,73	-	-
7	1,12	8,87	9,95
8	1,54	9,33	9,84
9	1,55	7,94	8,46
*Cement CPV			

**Fig. 4:**
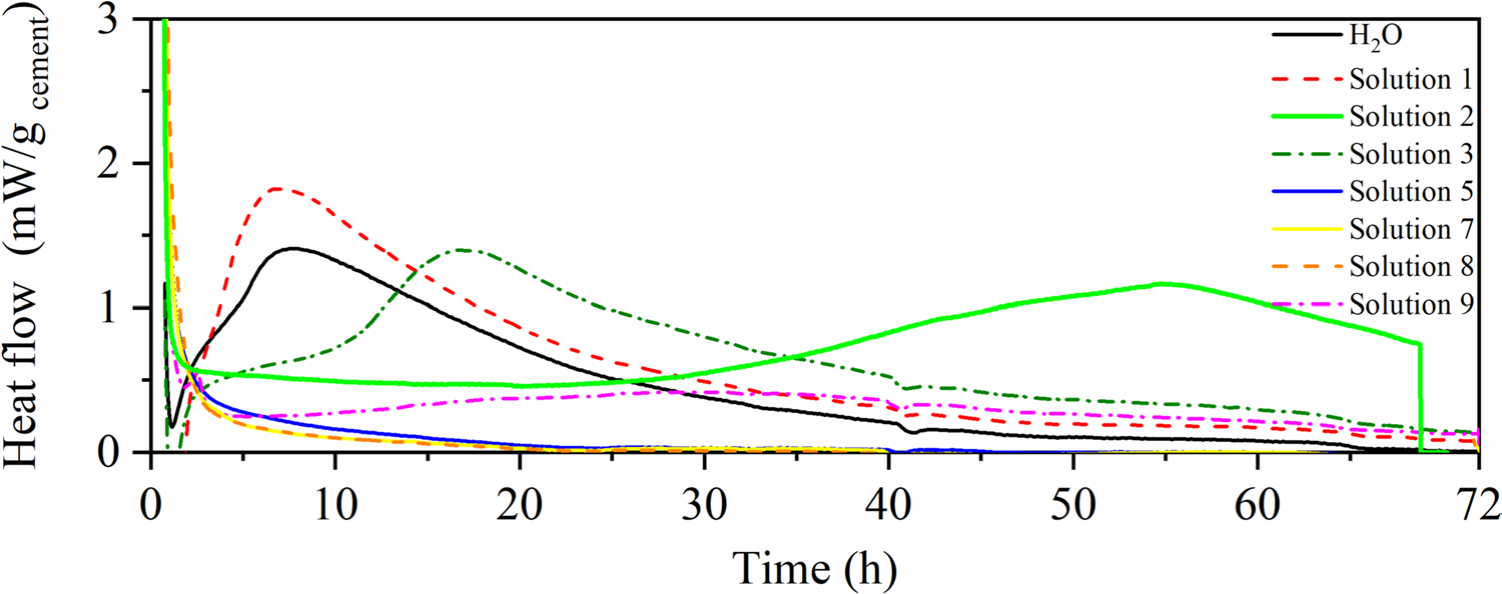
Calorimetry of solutions: Test 1 solution for analysis in calorimetry

Based on these preliminary results, solutions 1, 2, 3, 5, 7, 8, and 9 were selected for calorimetric analysis. These solutions, with pHs < 2, were pre-chosen due to the prolongation reported in the hydration kinetics curve in studies published in literature (Ramachandran and Grutzeck 1993; Singh et al. 2003; Wildling, Walter, and Double 1984). Figure 4 shows the calorimetry test carried out using the same solutions as in Test 1 (Tables 2 and 4), and the related parameters are listed in Table 5.

**Table 5:** Hydration parameters—Test 1 solution for calorimetry analysis

Parameters	H_2_O	Solution 1	Solution 2	Solution 3	Solution 5	Solution 7	Solution 8	Solution 9
Initial setting (h)	4,71	3,72	40,49	12,95	N. I	17,98	N. I	N. I
Final setting (h)	7,78	7,22	55,05	16,86	N. I	31,1	N. I	N. I
Duration (h)	3,07	3,5	14,56	3,91	N. I	13,12	N. I	N. I
Duration of induction period-LIP (h)	0,35	N. I	22,26	7,4	N. I	N. I	N. I	N. I
Maximum heat flow (mW/g _cement_)	1,41	1,81	1,18	1,39	N. I	0,41	N. I	N. I

Figure 4 and Table 5 present the resulting curves and kinetic parameters. Evaluation of these results, combined with the normative recommendation of 20 ± 2 h leaching duration (Table S1, Online Resource 2), indicated three key factors for solution assessment: (i) leachate composition, (ii) pH and metal solubility, and (iii) contact period.

In this context***,**** Solution 1 (pH 5.72****)*** displayed hydration kinetics comparable to water, confirming its inadequacy for leaching, as it failed to sufficiently extend the induction period.

*Solution 2 (pH 0.68)* markedly prolonged the LIP to 22 h, delaying C-S–H formation and extending the setting time beyond 23 h. This extended induction period, coupled with reduced nucleation and growth of hydrates, created conditions favorable for studying the release and transport of PTC (Kumar et al. 2012). Solution 2, therefore, emerged as the most promising medium for acidic leaching protocols representative of aggressive environmental or physiological conditions.

*Solution 3 (pH 0.89)* contained the same HNO₃ concentration but twice the NaOH concentration of Solution 2. Despite similar pH values, the calorimetric analysis revealed shorter LIP (7.4 h vs. 22.3 h), demonstrating that base concentration alters ionic strength, phase solubility, and hydration kinetics. This confirmed that Solution 2 was more effective in retarding hydration.

*Solution 7 (with isopropanol)* delayed the onset of hydration to ~ 18 h but failed to preserve the characteristic hydration peaks, unlike Solution 2. While not optimal under acidic conditions, isopropanol-based solutions may be relevant at higher pH levels for leaching metals that are stabilized in alkaline environments.

*Solutions 5, 8, and 9* had pH values comparable to those of Solutions 2 and 3, but they contained only acids, leading to degradation of the cementitious matrix. Their relevance requires validation by ICP-OES quantification of PTC release.

In summary, Solutions 2 and 3 met the chemical criteria for PTC mobilization (acid–base balance and acidic pH). However, Solution 2 offered superior leaching potential due to its prolonged induction period (22 h vs. 7.4 h) and extended setting time (> 20 h). Thus, Solution 2 was defined as the optimized leaching medium for subsequent methodological development.

Additional results for CPV cements (Tests 2 and 3; Online Resources 1 and 2, Figure S4, Table S9–S10) further confirmed these findings.

### Definition of the leaching stage and contact time: Application of the optimized solution to all samples

**Fig. 5:**
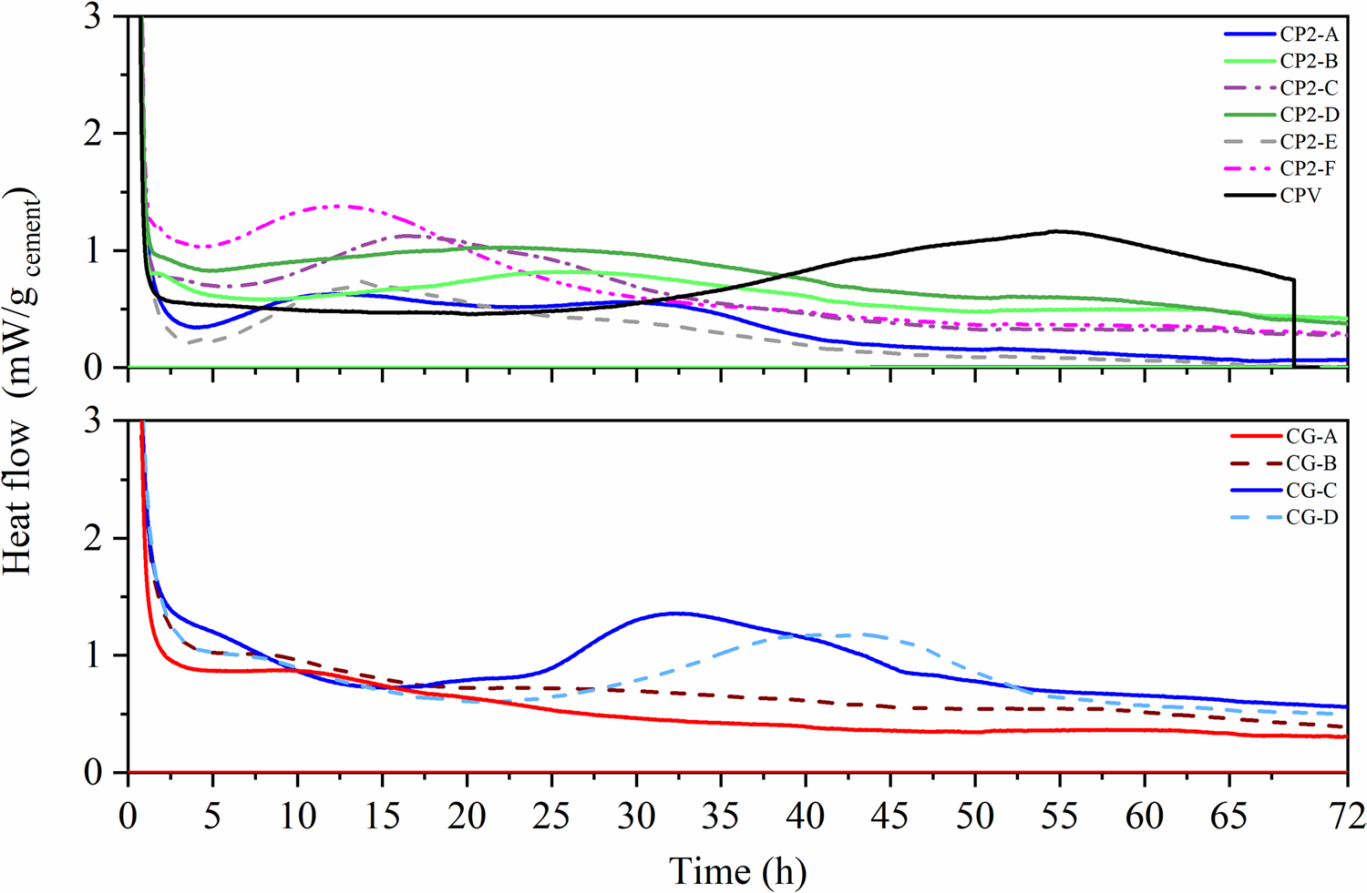
Hydration kinetics of cement with Solution 2 for calorimetry analysis

Figure 5 illustrates the reactivity curve of commercial cement when exposed to Solution 2. The hydration parameters obtained are shown in Table 6.

**Table 6:** Cement hydration parameters -Solution 2

Parameters	CP2-A	CP2-B	CP2-C	CP2-D	CP2-E	CP2-F	CPV	CG-A	CG-B	CG-C	CG-D
Initial setting (h)	8,21	20,31	12,15	15,57	9,56	9,44	40,49	-	-	25,8	34,9
Final setting (h)	10,74	26,17	16,15	24,48	13,66	12,65	55,05	-	-	32,93	44,23
Setting time (h)	3,32	5,86	4,01	8,92	4,11	3,22	14,56	-	-	7,13	9,33
Duration of induction period-LIP (h)	4,67	13,3	7,1	5,52	4,45	5,18	22,26	6,76	4,11	14,99	22
Maximum heat flow (mW/g _cement_)	0,63	0,81	1,12	1,02	0,7	1,37	1,18	0,87	1,01	1,31	1,19

Distinct kinetic behaviors were observed among the cements. Among the CP-II cement, CP2-D exhibited a late exothermic peak, high heat flowand an initial setting time exceeding 15 h, whereas CP2-A, B, and E displayed lower, prolonged heat flows, consistent with slower hydration. As expected, CPV cement exhibited a pronounced silicate hydration peak (1.18 mW/g) but maintained an extended induction period. Among type G cements, CG-C and CG-D exhibited delayed C₃S hydration peaks at ~ 30 h, whereas CG-A and CG-B showed consistently low heat flow, suggestive of decalcification.

The initial heat peak, associated with C₃A reaction with sulfates, was strong for all samples. However, the induction period was highly sensitive to C₃S polymorphism, C₂S content, and sulfate balance. CPV, CG-C, and CG-D had the most extended induction period (≥ 22 h), with subsequent silicate hydration and setting times exceeding 25 h. This behavior may be related, in part, to differences in the relative content of C₂S and C₃S and to possible variations in the polymorphism of C₃S, factors recognized in the literature as determinants of the initial reactivity of clinker. Furthermore, the balance between sulfates and C₃A may play a regulatory role in the kinetics during the first few hours, while the chemistry of the solution may inhibit the nucleation of products such as C-S–H, extending the induction period differently for each sample.

The prolonged induction period creates conditions favorable for leaching PTCs under near anhydrous or partially hydrated states. Moreover, differences between groups highlight the influence of mineralogical composition (C₃S, C₂S, C₃A, C₄AF), solution pH, and mineral additions (slag, pozzolan, limestone filler) on hydration kinetics.

Solution 2 reliably extended the induction period across cement types, ensuring a test window where PTC release can be evaluated prior to substantial hydrate formation. This supports its suitability as the leaching solution for the proposed methodology.

Given these parameters obtained in Fig. 5 illustrates the reactivity curve of commercial cement when exposed to Solution 2. The hydration parameters obtained are shown in Table 6, the most suitable stage for the leaching test is during the *induction period.* The justification for this choice is that, in the induction interval extended by the optimized solution, the precipitation of new hydrated phases occurs gradually, resulting in a plateau with a low heat release rate. It is inferred that there are still many free ions in the pore solution (Hewlett and Liska 2004; Taylor 1997).

The nucleation inhibition theory suggests that the interaction between ions and Al (OH) or Na^+^ alters the nucleation and growth of hydration products, making it the most suitable explanation for this prolongation phenomenon (Huang et al. 2022; Suraneni and Flatt 2015; Zhou et al. 2023). During LIP, the of potentially toxic íons compounds (PTC) remain free in the pore solution, increasing their detectability in ICP, especially when the pH is low, as demonstrated in a study (Wang et al. 2016). Wang et al (2016) demonstrated, using scanning electron microscopy (SEM), that when the pH is low (< 2), heavy metal ions generally adhere to the surface of cementitious materials and are more likely to leach out. When the pH was between 5 and 6, the heavy metal ions were inserted into the micropores of the cementitious materials, performing an isomorphous substitution due to the high C-S–H and Ca (OH)_2_ content. For the *contact period*, although each cement exhibited different kinetic behavior with the same solution, demonstrating the singularity of the analysis for each cement, a time of 19 ± 1 h was set, corresponding to the LIP as referred to in the CPV, for agitation between the sample and the leaching solution. However, for other cements, the contact time may vary depending on the LIP.

### Leaching test

Tables 7 and 8 show the results of the leaching tests. Table 7 shows the results of the quantitative leaching tests in accordance with standards NBR 10005 (ABNT 2004) and TCLP 1311 (USEPA 1992). Table 8 presents the quantitative results of the cements, according to the tests defined in Leaching Tests—TL.1.

**Table 7:** Leaching of Group 1 cements according to TCLP

Element	Unit	CP2-A.1		CP2-B.1		CP2-C.1		CP2-D.1		CP2-E.1		CP2-F.1		CPV.1		CG- A.1		CONAMA 396/08	NBR10004	USEPA, 2004
		Mean	± SD	Mean	± SD	Mean	± SD	Mean	± SD	Mean	± SD	Mean	± SD	Mean	± SD	Mean	± SD			
Ba	mg/L	0,688	0,0003	0,939	0,0721	-	-	0,217	0,0820	0,890	0,0021	0,842	0,0007	0,830	0,0035	0,953	0,0014	0,7	0,7	0,001
Cd	mg/L	-	-	-	-	0,058	0,0989	-	-	-	-	-	-	-	-	-	-	0,005	0,005	-
Co	mg/L	0,030	0,0003	0,030	0,0000	0,030	0,0007	0,030	0,0002	0,030	0,0001	0,030	0,0000	0,030	0,0001	0,033	0,0042	-	-	-
Cr	mg/L	0,136	0,0219	0,161	0,0276	0,102	0,0442	0,068	0,0689	0,111	0,0078	0,184	0,0007	0,177	0,0000	0,162	0,0042	0,05	0,05	-
Cu	mg/L	0,028	0,0029	0,033	0,0004	0,030	0,0007	0,032	0,0036	0,030	0,0001	0,030	0,0001	0,033	0,0003	0,033	0,0037	2	-	0,0013
Fe	mg/L	0,041	0,0001	0,037	0,0030	0,028	0,0015	0,032	0,0109	0,036	0,0006	0,034	0,0001	0,033	0,0004	0,037	0,0030	0,3	-	-
La	mg/L	0,209	0,1336	-	-	-	-	-	-	-	-	-	-	-	-	-	-	-	-	-
Mn	mg/L	0,001	0,0001	-	-	-	-	0,001	0,0008	0,001	0,0001	0,001	0,0000	0,001	0,0001	0,001	0,0004	0,1	-	0,05
Mo	mg/L	0,050	0,0045	0,312	0,0502	0,074	0,0211	0,177	0,2063	0,047	0,0010	0,044	0,0003	0,343	0,0007	0,039	0,0004	0,07	-	-
Ni	mg/L	0,033	0,0003	0,033	0,0000	0,033	0,0001	0,033	0,0006	0,033	0,0001	0,033	0,0001	0,033	0,0000	0,040	0,0103	0,02	-	-
Pb	mg/L	-	-	-	-	-	-	-	-	-	-	-	-	-	-	-	-	0,01	0,01	-
Sb	mg/L	0,045	0,0039	0,045	0,0075	0,032	0,0082	0,032	0,0079	0,038	0,0066	0,040	0,0005	0,041	0,0052	0,050	0,0021	-	-	0,0056
Sc	mg/L	0,758	0,0566	0,822	0,0658	0,614	0,0071	0,294	0,0877	0,433	0,0000	0,727	0,0000	0,659	0,0035	0,672	0,0028	-	-	-
Ti	mg/L	0,070	0,0115	0,065	0,0137	0,041	0,0279	0,020	0,0262	0,048	0,0035	0,063	0,0000	0,062	0,0001	0,061	0,0001	-	-	-
V	mg/L	-	-	-	-	-	-	-	-	-	-	-	-	-	-	-	-	0,05	-	-
Zn	mg/L	0,006	0,0022	0,003	0,0006	0,002	0,0006	0,002	0,0026	0,004	0,0004	0,002	0,0001	0,002	0,0000	0,005	0,0003	5	-	-

**Table 8:** Leaching of cement according to the optimized TL.1 method

		**CP2-A.1**		**CP2-B.1**		**CP2-C.1**		**CP2-D.1**		**CP2-E.1**		**CP2-F.1**		**CPV-1**		**CG-1**		**CONAMA 396/08**	**NBR 1004**	**USEPA, **2004
Element	Unit	Mean	± SD	Mean	± SD	Mean	± SD	Mean	± SD	Mean	± SD	Mean	± SD	Mean	± SD	Mean	± SD			
Al	mg/L	0,26	0,195	0,12	0,001	0,15	0,000	0,07	0,015	0,14	0,006	0,10	0,013	0,06	0,000	0,06	0,002	0,2	-	-
B	mg/L	-	-	-	-	-	-	-	-	-	-	-	-	-	-	-	-	0,5	-	-
Ba	mg/L	1,41	0,424	1,47	0,021	3,09	0,445	0,22	0,374	2,63	0,141	0,92	0,212	-	-	-	-	0,7	0,70	0,001
Bi	mg/L	0,02	0,006	0,01	0,004	0,02	0,008	0,03	0,019	0,02	0,001	0,02	0,002	0,02	0,008	0,02	0,003	-	-	-
Cd	mg/L	-	-	-	-	-	-	-	-	-	-	-	-	-	-	-	-	0,005	0,005	-
Co	mg/L	-	-	-	-	-	-	-	-	-	-	-	-	-	-	-	-	0	0	-
Cr	mg/L	0,27	0,072	0,33	0,022	0,70	0,006	-	-	0,29	0,013	0,19	0,093	-	-	-	-	0,05	0,05	
Cu	mg/L	-	-	-	-	-	-	-	-	-	-	-	-	-	-	-	-	2	-	0,0013
Ga	mg/L	-	-	-	-	-	-	-	-	-	-	-	-	-	-	-	-	-	-	-
In	mg/L	0,11	0,012	0,11	0,003	0,11	0,002	-		0,11	0,003	0,11	0,001	-		-		-	-	-
La	mg/L	0,16	0,055	0,23	0,008	0,26	0,006	0,46	0,148	0,22	0,018	0,15	0,036	0,57	0,014	0,56	0,001	-	-	-
Li	mg/L	-	-	0,47	0,003	-		-		-		0,25	0,029	-	-	-	-	-	-	-
Mg	mg/L	-	-	-	-	-	-	-	-	-	-	-	-	-	-	-	-	-	-	-
Mn	mg/L	0,00	0,001	0,00	0,000	0,00	0,000	0,00	0,000	0,00	0,000	0,00		0,00		0,00		0,1	-	0,05
Ni	mg/L	-	-	-	-	-	-	-	-	-	-	-	-	-	-	-	-	0,02	-	-
Pb	mg/L	-	-	-	-	-	-	-	-	-	-	-	-	-	-	-	-	0,01	0,01	-
Sb	mg/L	2,64	0,757	3,31	0,113	3,83	0,064	3,11	0,354	3,40	0,184	2,22	0,523	3,55	0,346	3,29	0,021	0,005	-	0,0056
Sr	mg/L	7,48	0,368	30,40	0,141	10,55	0,636	3,08	4,522	9,12	1,153	16,60	3,253	-		-		-	-	-
Tl	mg/L	20,70	3,253	25,05	0,495	26,20	0,424	8,77	4,851	24,45	0,778	19,65	2,333	5,49	0,269	5,30	0,035	-	-	-
V	mg/L	0,01	0,035	0,03	0,007	0,07	0,003	0,30	0,156	0,04	0,009	-	-	0,41	0,004	0,41	0,003	0,05	-	-
Zn	mg/L	-	-	-	-	-	-	-	-	-	-	-	-	-	-	-	-	5	-	-

TCLP analysis detected Ba, Cd, Co, Cr, Cu, Mn, Mo, Ni, Sb, Sc, Ti, and Zn, whereas the proposed TL.1 method identified Al, Ba, B, Bi, Cr, La, Li, Mn, Sb, Sr, Tl, and V. Notably, Cd, Co, Cu, Mo, and Ni appeared only in TCLP, likely due to differences in calibration standards or analytical sensitivity. Conversely, B, Bi, La, Li, Sr, Tl, and V were detected only in TL.1, emphasizing the role of method-specific standards and potential interferences (Table 7).

When compared to environmental regulations, TCLP indicated that Ba, Cd (C2P-C), Cr, and Mo exceeded CONAMA 396/08 limits. According to USEPA (2004), Ba, Cu, and Sb were above the limits/thresholds. In TL.1, Al (CP2-C.1), Ba, Cr, Sb, and V exceeded CONAMA values, and NBR 10004. For USEPA, Ba and Sb exceeded limits. In G cement, Sb and V also surpassed CONAMA limits (Tables 7 and 8).

The two methods yielded striking discrepancies. For Ba, TL.1 leached ~ 3 times more (0.922–3.083 mg/L) than TCLP (0.688–0.953 mg/L). For Cr, TL.1 released ~ 1.5 times more (0.190–0.266 mg/L) than TCLP (0.068–0.177 mg/L). The most dramatic case was V: TL.1 detected ~ 70 times more (2.22–3.55 mg/L) compared to TCLP (0.032–0.050 mg/L).

These differences confirm that leaching outcomes are highly sensitive to experimental conditions. Parameters such as pH, solid/liquid ratio (S/L), reagent composition, and leaching duration directly govern the mobility of potentially toxic compounds (PTCs). More acidic or alkaline media enhance solubility and mobility, increasing leaching (Li et al. 2001; Malviya and Chaudhary 2006). Moreover, reagent selection can shift hydration kinetics, forming new hydrated phases that either encapsulate or destabilize metals, altering their leachability (Navarro et al. 2007; Tsai and Tsai 1998). Overall, the proposed method produced higher PTC release than TCLP, demonstrating the importance of method design in capturing realistic leaching scenarios.

The leaching results are presented as mean ± standard deviation (SD), allowing assessment of variability among replicates. In general, most analytes showed low to moderate dispersion, with a coefficient of variation (CV = SD/mean × 100) below 20%, a value adopted in this study as indicative of acceptable repeatability. Values below 10% were considered low dispersion, values between 10 and 20% were considered moderate dispersion, and values above 20% were considered high dispersion.

The highest SD values were observed mainly for Tl and Sr. For Tl, the most notable results were 20.70 ± 3.253 mg/L (CP2-A.1), 8.77 ± 4.851 mg/L (CP2-D.1), and 19.65 ± 2.333 mg/L; and for Sr, 7.48 ± 3.368 mg/L, 3.08 ± 4.522 mg/L, and 16.60 ± 3.253 mg/L. These results suggest greater variability under some extraction conditions, probably due to matrix heterogeneity. Even so, this variability does not compromise the overall interpretation of the data, since, for most analytes, the differences observed between methods were greater than the variability among replicates within the same method. Only cases with high relative dispersion, such as Sr and Tl in CP2-D.1, should be interpreted with caution.

In addition, to ensure the analytical reliability of the data, the calibration curve was constructed using eight points from a multielement standard solution at different concentrations, resulting in a linear correlation coefficient (R) greater than 0.99.

To structure the quantitative discussion, Tables 9 and 10 reorganizes the results by method (TCLP, TL.1, TL.2,TL.3) and groups the detected elements as amphoteric, oxyanion-forming, or cationic oxides.

**Table 9:** Comparison of PTC determined by different methods-Part 1

Element	Unit	CP5. TCLP	CP5.1		CP5.2		CP5.3		CP2-B TCLP	CP2-B.1		CP2-B.2		CP2-B.3		CP2-D TCLP	CP2-D.1		CP2-D.2		CP2-D.3	
			Mean	± SD	Mean	± SD	Mean	± SD		Mean	± SD	Mean	± SD	Mean	± SD		Mean	± SD	Mean	± SD	Mean	± SD
Al	mg/L	-	0,06	0,001	0,107	0,015	0,182	0,08	-	0,125	0,001	0,111	0,007	0,248	0,086	-	0,071	0,015	0,125	0,009	0,177	0,016
B	mg/L	-	-	-	-	-	-	-	-	-	-	-	-	0,026	0,005	-	-	-	-	-	0,042	0,009
Ba	mg/L	0.829	-	-	0,829	0,313	1,53	0,481	0.939	1,465	0,021	0,757	0,161	2,055	0,276	0,217	0,219	0,374	0,989	0,144	1,095	0,078
Bi	mg/L	-	0,021	0,011	0,017	0,001	0,02	0,003	-	0,014	0,004	0,016	0,004	-	-	-	0,031	0,019	0,029	0,002	0,004	0,002
Cd	mg/L	-	-	-	-	-	-	-	-	-	-	-	-	-	-	-	-	-	-	-	-	-
Co	mg/L	0,03	-	-	-		-		0.0300	-	-	-		-		0,03	-	-	-		-	-
Cr	mg/L	0,177	-	-	0,264	0,023	0,193	0,029	0.161	0,333	0,022	0,089	0,056	0,113	0,011	0,068	-	-	0,084	0,027	0,102	0,033
Cu	mg/L	0,033	-	-	-	-	-	-	0.0330	-	-	-	-	-	-	0,032	-	-	-	-	-	-
Ga	mg/L	-	-	-	-	-	-	-	-	-	-	-	-	-	-	-	-	-	-	-	-	-
In	mg/L	-	-	-	0,104	0,004	0,115	0,002	-	0,111	0,003	0,109	0,003	0,107	0,004	-	0,462	0,148	0,11	0,006	0,113	0,002
La	mg/L	-	0,56	0	0,126	0,059	0,205	0,027	-	0,233	0,008	0,14	0,029	0,249	0,035	-	-	-	0,156	0,013	0,253	0,016
Li	mg/L	-	-		0,832	0,017	1,042	0,082	-	0,472	0,003	0,654	0,032	0,516	0,042	-	0	0	0,04	0,068	-	-
Mg	mg/L	-	-	-	-	-	-	-	-	-	-	-	-	-	-	-	-	-	-	-	-	-
Mn	mg/L	-	-	-	-	-	0,001	0	-	0,001	0	-	0	0,001		-	-	-	-		-	
Mo		0.342							0,312							0,177						
Ni	mg/L	0,033	-	-	-	-	-	-	0,033	-	-	-	-	-	-	0,033	-	-	-	-	-	-
Pb	mg/L	-	-	-	-	-	-	-	-	-	-	-	-	-	-	-	-	-	-	-	-	-
Sb	mg/L	0,041	3,235	0,007	1,97	0,537	2,68	0,438	0,045	3,31	0,113	2,095	0,403	3,19	0,184	0,032	3,11	0,354	2,42	0,17	3,315	0,035
Sr	mg/L	-	-	-	15,85	5,445	15,8	0,849	-	30,4	0,141	13,6	1,414	17,3	2,121	-	3,083	4,522	11,75	1,909	13,5	0,566
Sc		0,658							0.822							0.294						
Tl	mg/L	-	5,255	0,007	17,75	3,041	19,3	1,697	-	25,05	0,495	17,6	1,414	22,5	1,273	-	8,77	4,851	18,15	1,202	22,85	0,354
Ti		0,062							0.0648							0,02						
V	mg/L	-	0,407	0,001	-	-	0,019	0,027	-	0,035	0,007	-	-	0,073	0	-	0,303	0,156	-	-	0,083	0,001
Zn	mg/L	0,002	-	-	-	-	-	-	0,003	-	-	-	-	-	-	0,002	-	-	-	-	-	-

**Table 10:** Comparison of PTC determined by different methods-Part 2

Element	Unit	CG. D TCLP	GC. D −1		GC. D −2		GC. D −3	
			Mean	± SD	Mean	± SD	Mean	± SD
Al	mg/L	-	0,057	0,002	0,108	0,003	0,216	0,007
B	mg/L	-	-	-	-	-	-	-
Ba	mg/L	0.551	-	-	0,209	0,028	0,270	0,015
Bi	mg/L	-	0,017	0,001	0,021	0,009	0,005	0,004
Cd	mg/L	-	-	-	-	-	-	-
Co	mg/L	0.0302	-	-	-	-	-	-
Cr	mg/L	0.0197	-	-	0,022	0,010	0,105	0,008
Cu	mg/L	0.0295	-	-	-	-	-	-
Ga	mg/L	-	-	-	-	-	-	-
In	mg/L	-			0,114	0,002	0,105	0,002
La	mg/L	-	0,563	0,001	0,139	0,010	0,256	0,023
Li	mg/L	-	-	-	-	-	-	-
Mg	mg/L	-	-	-	-	-	-	-
Mn	mg/L	-	0,000	0,000	-	-	-	-
Mo		0,032						
Ni	mg/L	0,033	-	-	-	-	-	-
Pb	mg/L	-	-	-	-	-	-	-
Sb	mg/L	-	3,250	0,014	2,035	0,177	3,130	0,255
Sr	mg/L	-	-	-	13,900	2,121	20,350	0,778
Sc		0.704						
Tl	mg/L	-	5,270	0,014	16,900	1,273	22,800	0,707
Ti		0,066						
V	mg/L	-	0,407	0,001	-		0,062	0,020
Zn	mg/L	0,003	-	-	-	-	-	-

The leaching results are presented as mean ± SD to document the variability among replicates. In Table 9, the highest SD values were mainly associated with Sr and Tl, especially in CP5.2 and CP2-D.1. The most pronounced dispersion was observed for Sr in CP2-D.1 (3.083 ± 4.522 mg/L) and Tl in CP2-D.1 (8.770 ± 4.851 mg/L), indicating lower repeatability under this extraction condition. Even so, for most analytes, the SD remained small (CV < 10%) relative to the overall differences observed between the methods, reaffirming the main comparative trends.

Based on the Table 9, the quantitative discussion will be divided into subgroups: Amphoteric, Anionic, and Cationic oxides.

#### Amphoteric oxides

In the TCLP procedure, the detection of antimony (Sb) was significantly lower than in the TL.1, TL.2, and TL.3 tests, due to pH conditions, hydration kinetics, and the S/L ratio. In the TCLP, the Sb concentration was 0.0412 mg/L, while in the other tests there was a substantial increase, ranging from 48 to 86 times, with the highest levels observed at extreme pH values. These results are explained by the amphoteric nature (pH < 7 and pH > 9) of Sb. Aluminum (Al) showed higher detection in TL.2 and TL.3, also suggesting pH-dependent amphoteric solubility and differences in retention by the cementitious matrix. Copper (Cu) and zinc (Zn) were detected only in the TCLP, which may reflect differences in aqueous speciation and retention by the matrix under the distinct pH conditions of the leaching methods. Chromium (Cr) showed variable release among the methods, likely due to changes in speciation dependent on redox reactions, especially the reduction of the more soluble Cr⁶⁺ to the less mobile Cr^3^⁺ (Chaudhari and Biernacki, 2017). Therefore, the release of amphoteric elements appears to be controlled by the combined effects of pH, speciation, and interaction with hydration products, and not solely by pH alone (Möschner et al. 2009) (Table S7—Online Resource 2).

#### Oxyanions

Oxyanion release generally peaked at pH 8–10, with reduced solubility at pH 4–6 and > 11, except for B, which did not follow this trend. Mo and Ni were detected only by TCLP, explained by the absence of Mo standards in TL.1 calibration and Ni levels below the detection limit. Vanadium (V) showed a distinct pH-dependent behavior: it was not detected in the highly alkaline TCLP and TL.2 solutions (pH 10.53), but it was detected in TL.1 (pH 6.32) and TL.3 (pH 8.29). This suggests that V release was influenced by pH-dependent speciation and by the affinity of vanadium oxyanions for the cementitious matrix. Under intermediate pH conditions, V may remain in more mobile aqueous forms, whereas under more alkaline conditions its mobility may decrease due to stronger retention by hydration products, consistent with previous observations by Engelsen et al. (2010). Figure S6 in Online Resource 1 helps visualize the points discussed.

#### Metal cations

Cationic metals exhibit a leaching pattern in more acidic environments (pH < 7). The cationic elements (Ba, Bi, Co, La, Li, Sr, Sc, Tl, Ti) were leached more efficiently in TL.1–TL.3 than in TCLP. The proposed methods increased solubility by adjusting the S/L ratio, stirring intensity, pH, and reagent chemistry, thereby modifying the cement hydration kinetics. The prolongation of induction and set times, as well as the alteration of the hydration curves, favored greater metal release. Under these conditions, the delayed formation of hydration products likely reduced the retention capacity of the cementitious matrix, increasing the mobility of cationic species in solutions. In contrast, the TCLP (pH 3–5) showed lower values, likely due to the encapsulation of metals in C-S–H and ettringite (Fig. 7S – Online Resource 1), as well as more intense retention by the newly formed hydrates.

In summary, the release of PTCs in anhydrous cement is mainly controlled by the interaction between the mineralogical composition of the matrix and the chemical conditions of the extracting medium, especially pH. Under strongly acidic conditions, a high degree of dissolution of the most reactive cement phases occurs. This process directly influences the solubility and mobility of potentially toxic elements. Thus, the observed detection depends not only on the total concentration of the elements in the cement, but also on their association with specific mineral phases and on their response to the imposed extraction conditions.

### Quantification of compounds in solid matrix

**Fig. 6:**
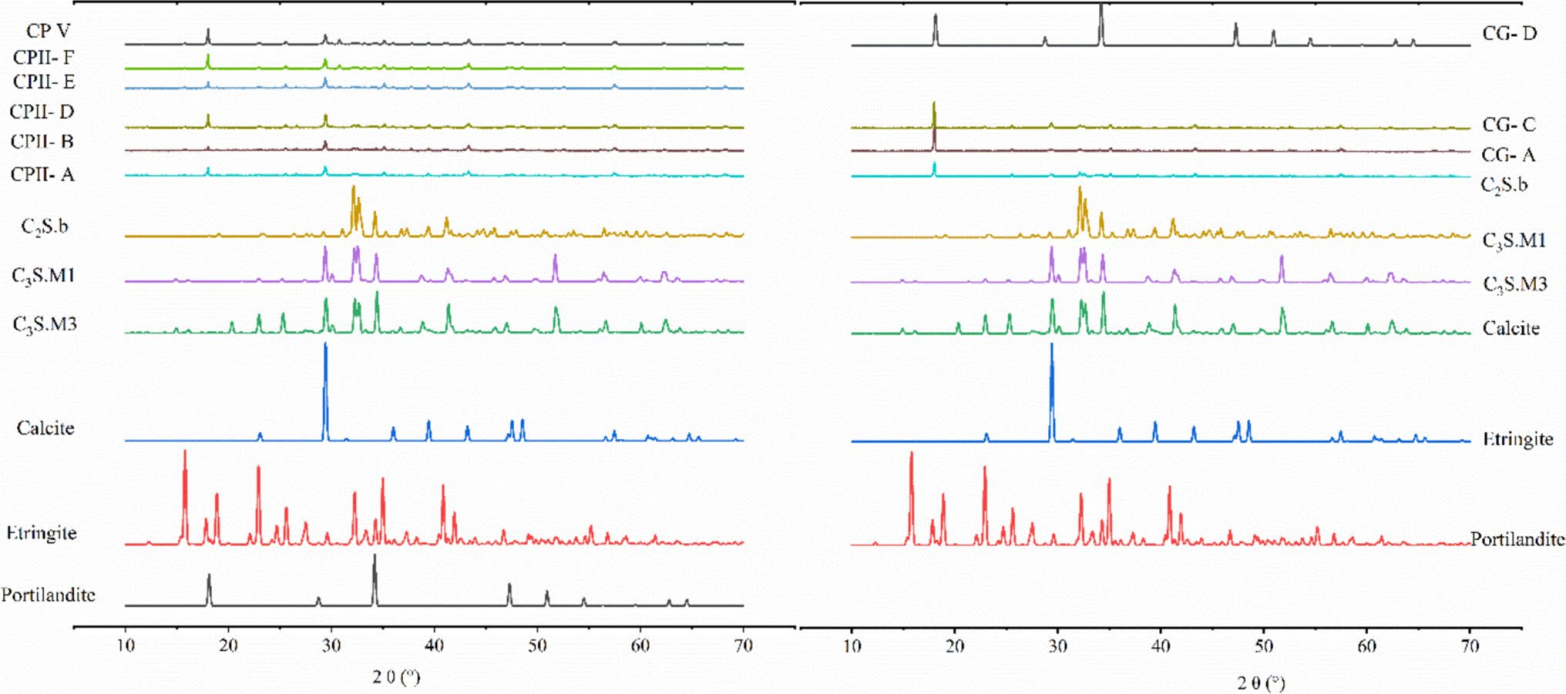
Diffractogram of the leached solid sample

Tests carried out with HNO₃/NaOH solution (pH 0.68) caused significant changes in the mineralogical composition of anhydrous cement, as observed through quantitative X-ray diffraction (XRD) analyses (Table 11 and Fig. 6).

**Table 11:** Phase assemblage of leached solids determined by XRD/Rietveld analysis, expressed as wt.%

	CP2-A	CP2-B	CP2-D	CP2-E	CP2-F	CPV	CG-A	CG-C	CG-D
Belite (C_2_S.b)	9,334	6,689	6,850	4,747	3,180	12,818	13,504	7,835	12,668
Alite (C_3_S.M1)	0,000	0,000	0,000	0,000	0,000	0,000	0,000	0,000	5,929
Alite (C_3_S.M3)	8,088	10,261	10,975	0,000	8,111	5,906	17,421	0,000	0,000
Dolomite	0,000	0,000	0,000	0,679	9,436	1,088	0,000	0,132	0,000
Ettringite	7,144	5,141	5,291	5,842	4,443	8,357	5,832	4,487	10,678
Portlandite	0,000	1,229	0,963	0,000	2,710	4,510	2,515	4,746	3,630
Calcite	0,595	21,387	27,848	20,399	20,401	11,147	3,168	3,241	11,979
Quartz	4,050	0,000	2,735	0,000	0,000	0,889	0,207	0,124	0,806
Anhydrite	0,000	0,007	0,000	0,000	0,000	0,000	0,000	0,000	0,000
Tobermorite	14,856	4,684	5,429	0,000	3,406	7,850	4,968	10,076	7,887
Amorphous fraction	55,933	50,610	39,908	50,850	48,313	47,437	52,385	69,360	46,423

XRD analysis of leached solids (40 days) revealed marked differences among CP2, CG, and CPV cements compared to their anhydrous counterparts. Anhydrous phases (C₃S, C₂S) persisted, confirming that the acidic medium delayed hydration. CP2 cements showed reduced C₂S, while CPV and CG-C maintained higher C₂S, C₃S, portlandite, and tobermorite contents, consistent with their extended induction periods.

Leaching promoted Ca^2^⁺ mobilization, evidenced by increased carbonate phases (calcite up to 28%), attributed to Ca reprecipitation with atmospheric CO₂ during drying. Secondary phases such as ettringite (≤ 10%) and tobermorite (≤ 14.8%) indicated partial rehydration and nucleation of ordered C-S–H structures even under acidic exposure. Portlandite was largely dissolved (< 1–4%), except in CPV and CG-C.

A substantial rise in the amorphous fraction (40–69%) confirmed structural disorganization and selective dissolution of crystalline phases. Comparisons with hydrated cement from the literature highlight compositional differences, notably lower calcium content due to carbonate and tobermorite formation (Costa et al. 2021).

Overall, results demonstrate that the methodology effectively extended the hydration induction period, enabling PTC mobilization under controlled conditions. Mineralogical evidence supports a mechanism of selective phase dissolution, Ca^2^⁺ reprecipitation as carbonates, and secondary hydrate formation, linking mineralogical evolution to hydration kinetics in aggressive environments (De S. da Paixão et al. 2025; Costa et al. 2021).

The XRD results not only confirm the prolongation of the induction period but also provide important evidence on the mechanisms controlling the mobilization and retention of potentially toxic compounds (PTCs) during leaching. The persistence of anhydrous phases, such as C₃S and C₂S, indicates that the acidic environment delayed hydration and, consequently, limited the initial formation of hydrated products capable of incorporating or encapsulating these elements. On the other hand, the marked reduction in portlandite indicates matrix decalcification and loss of buffering capacity, creating conditions more favorable to selective dissolution and the release of species associated with the more soluble phases. Thus, the mineralogical data suggest that the proposed leaching method results in greater release of PTCs into the pore solution.

### Proposal for a quantitative method for the ptcs of anhydrous cement

**Fig. 7:**
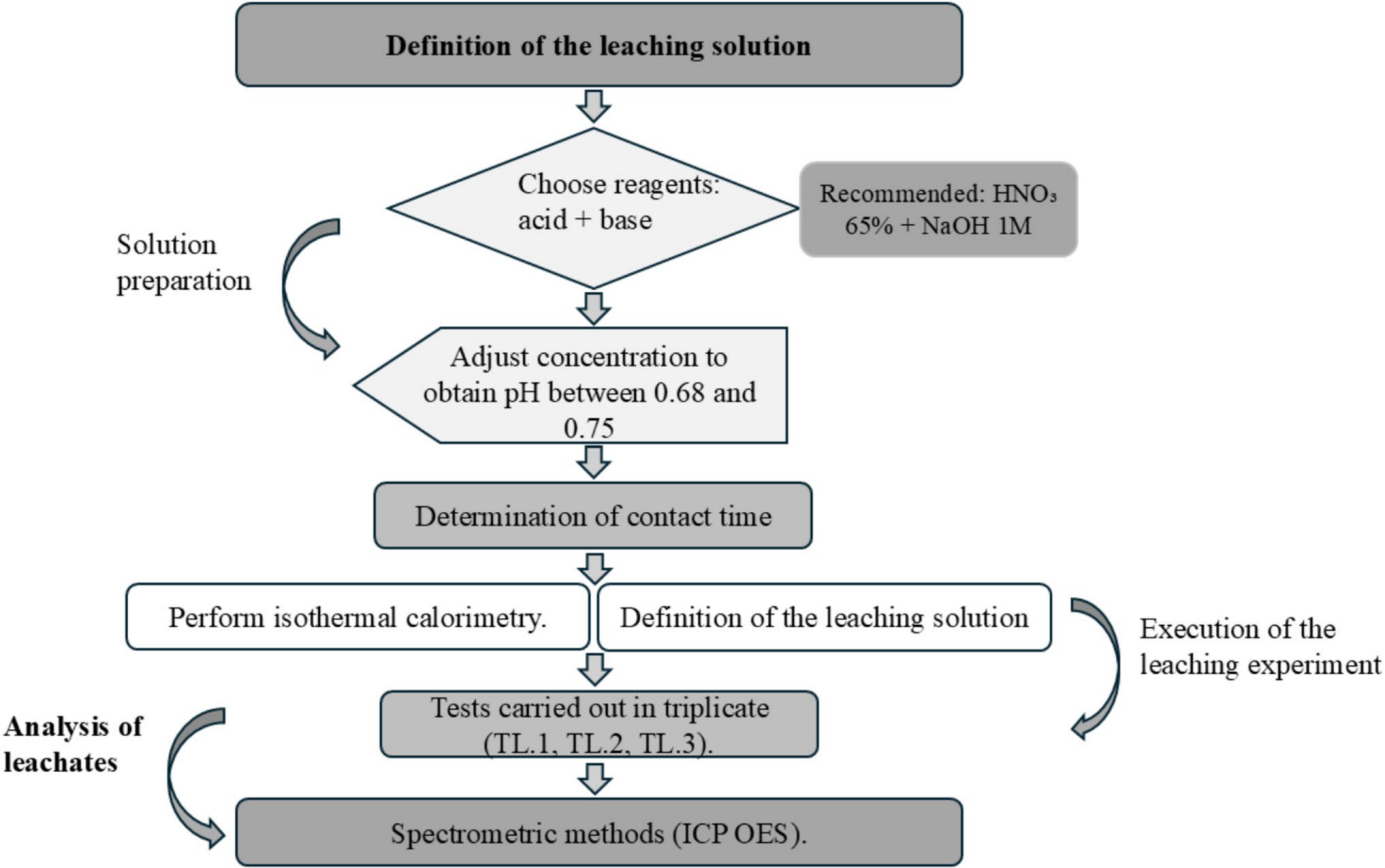
Flowchart of methodology for anhydrous cement leaching

The developed method quantifies Potentially Toxic Compounds (PTC) in anhydrous cement by leaching. The leaching solution is defined with pH adjusted between 0.68 and 0.75 (NaOH + HNO₃). Isothermal calorimetry is then used to determine the optimal contact time, defined by the LIP method. Tests are carried out in triplicate, and leachates are analyzed by ICP OES, with validation through external calibration, LOD, and LOQ. The methodology is compared to TCLP, differing by the extension of the LIP. Its aim is to establish a specific procedure to assess PTCs release and maximum solubility in critical scenarios, considering potential impacts on human health and the environment (Fig. 7).

Based on the tests carried out in the previous stages, a method was developed for the quantification of Potentially Toxic Compounds (PTCs) by leaching in anhydrous cement.

#### Preparation of the solution

For the development of the method, the first step is to define the solution for the leaching tests. The selected solution was proposed with the aim of prolonging the induction period. From this perspective, the intention is for the solution to meet the need to extend the induction period of the hydration stage, ensuring that hydrates are formed only after a minimum period of 20 h, the time established to allow equilibrium between the sample and the leaching solution.

The pH recommended in this methodology to prolong the induction stage is between 0.65 and 0.70 (suggested composition: 0.005 mol/L NaOH and 0.03 mol/L HNO₃). The pH range of 0.65–0.70 was selected because pH is a fundamental parameter in controlling leaching, directly influencing the solubility of elements in cementitious matrices. Since the natural pH of aqueous cement suspensions is high due to calcium release, it was necessary to adopt a strongly acidic condition to promote phase dissolution and element release. pH-dependent leaching studies, such as EPA Method 1313, show that the mobilization of several elements increases under acidic conditions. Thus, this range was adopted because it represents severe, controlled, and reproducible extraction conditions. This justification is also supported by Figure S3, adapted from EPA Method 1313, which illustrates the dependence of metal solubility on pH.

However, it is essential to combine an acid and a base, as using an acid alone can alter the hydration curve and lead to degradation. Additionally, reagents such as sulfuric acid (H₂SO₄) are not recommended, as they alter the hydration curve even when combined with a base. The suggestion is to use reagents such as nitric acid (HNO₃, 65%) and sodium hydroxide (NaOH, 1 M), which are justified by their compatibility with ICP measurements.

In addition, the solution should have a proportion corresponding to 10 times the sample mass (S: L = 1:10). This determination was established and justified in detail in the corresponding section.

#### Stirring of the solid with the extracting solution

Samples must be stirred at a speed of 14,000 revolutions per minute (rpm) for 1 min, followed by an increase to 21,000 revolutions per minute for an additional minute, totaling a mixing period of 2 min. After this step, tests must be performed to measure the post-mixing pH values.

#### Exothermic calorimetry

After stirring the samples with the solutions, an amount of 6 to 7 g of the resulting mixture is transferred to ampoules, which are then placed in a calorimeter to carry out the test over a period of 72 h.

During the analysis of the obtained results, it is crucial to verify whether the solution with the selected pH effectively prolonged the induction period without compromising the hydration curve. If any alteration in the hydration kinetics curve is identified, the pH of the solution must be adjusted.

#### Leaching method

Based on the results obtained for the appropriate solution in the calorimetry tests, three similar solutions for each sample will be prepared in 250 mL beakers previously decontaminated with HNO₃ (follow Sect. 2.2.4). The amounts of HNO₃ and NaOH must be determined in preliminary screening tests conducted prior to the leaching tests.

The L/S ratio must be 10 ± 0.3 mL/g (10:1), and the suspensions must be subjected to stirring for 19 ± 1 h. The pH must be measured before filtration. The leaching procedure is then divided into three stages:

In Test 1, the samples are mixed directly in the beaker using circular movements. In Tests 2 and 3, the samples are stirred at 14,000 rpm for 1 min and then at 21,000 rpm for an additional 1 min, totaling 2 min of mixing. In all tests, the post-mixing pH values were measured. Only in Test 3, must the samples be re-acidified after mixing with 50% nitric acid, so that each sample reaches 2% acidification.

Subsequently, all mixtures must be mechanically stirred in a mixer at 230 rpm for 19 h at 25 °C. Finally, after this period, the mixture is filtered using a syringe filter and transferred to Falcon tubes. The samples will be kept refrigerated at 4 °C until analysis by ICP-OES (9800 Series, Shimadzu).

It is recommended that the experiment be carried out in duplicate or triplicate for the quantitative analysis of leaching. If performed in duplicate, each sample should be analyzed twice. The proposed methods for the spectrometric analysis employed (ICP-OES) were validated according to the external calibration technique, based on the analytical parameters, the limit of detection (LOD) (3σ/s), and the limit of quantification (LOQ) (Table S10 – Online Resource 2).

It should be considered as a limitation that the proposed methodology evaluates the release of elements under a controlled extraction condition, representative of a critical solubilization scenario. Therefore, the results do not necessarily correspond to the behavior of the material under all real exposure conditions, but rather to its maximum release potential under circumstances favorable to the mobilization of its constituents. Even so, the method can be applied in comparative studies among different types of cement, in preliminary environmental and occupational risk assessment, in the screening of materials regarding their potential to release potentially toxic constituents, and as a supporting tool for the development of formulations and management strategies in the cement industry.

#### Safety

Regarding precautions related to beaker contamination, decontamination procedures using 10% diluted nitric acid should be applied 24 h before carrying out the tests.

#### Reagents and standards

Extraction solution: add 1.0 N NaOH and 65% HNO₃.

#### Preparation method

Measure pH before leaching, during the first 15 min, after stopping the horizontal shaker (without filtration), and after acidification.

##### Note

pH measurement must be performed within 15 min after obtaining the eluate, that is, the solid + liquid mixture after leaching, to avoid solution neutralization after exposure to CO₂ or after filtration.

##### Retained material

The solid fraction should be dried in an oven at a temperature up to 38 °C. Subsequently, it should be homogenized in a mill and stored in a desiccator until the necessary chemical analyses are carried out. XRD and XRF analyses are recommended.

#### ICP-OES calibration

For calibration, if a multielement calibration standard is used (Table S11), it is not necessary to prepare multiple separate curves in volumetric flasks to avoid interferences. Instead, prepare an 8-point calibration curve by varying the concentration of the standard solution and diluting it in the appropriate volumetric flask.

The newly proposed methodology aims to establish a procedure for leaching anhydrous cement, considering the impact of cement particles on human health and the environment. From this perspective, anhydrous cement may undergo leaching, releasing potentially toxic substances. This methodology enables detection of PTCs and their maximum solubilization under the most critical scenario.

## Conclusions

Given the inefficiency of existing standards for leaching quantities in anhydrous cement, this research aimed to propose a new method for quantifying potentially toxic compounds in anhydrous cement. Based on the results obtained, the main conclusions were:

The results indicated that the most suitable leaching solution for analyzing anhydrous cement should comprise a specific proportion of nitric acid and sodium hydroxide, with a pH between 0.65 and 0.7. The hydration kinetics of the different types of cement required specific adjustments in the formulation of the leaching solution for each material. It was observed that the type of polymorph present significantly influenced the hydration of the samples, the proportion of C₃S, the presence of C₂S, and the sulphate balance. Among the process stages, the induction period was the most favorable for leaching. Thermal analysis by calorimetry was imperative for optimizing the contact time, maximizing the efficiency of PTCs leaching, and highlighting the need to adapt the method according to the specific composition of each cement.

The leaching tests indicated that the optimized solution contributed to the detection of 11 heavy metals compared with the conventional TCLP method. The proposed methodological refinement enabled the release and measurement of higher concentrations of the evaluated elements, indicating greater leaching efficiency under the tested conditions. Parameters such as pH, reagents, contact time, agitation, and the S/L ratio were decisive in modifying the hydration kinetics of the cement, prolonging the induction period and increasing the mobilization of elements during leaching.

In contrast, the TCLP method, with a pH range of 3 to 5, resulted in lower detection of elements, as hydrated phases formed that encapsulated the toxic compounds. These results highlight the relevance of the proposed new leaching method. Adjusting physicochemical parameters makes it possible to optimize the solubility of the elements. Furthermore, the method contributes to mitigating environmental impacts, protecting workers’ health, and enhancing the sustainability of the cement industry, especially in light of the growing use of waste co-processing.

The method developed proved effective in optimizing the detection of PTCs, including Ba, Cr, Cu, Zn, Pb, Ni, La, V, Co, Sb, Mo, and Sn. Based on the results obtained, further studies are recommended to investigate the reduction of pH during the leaching test, which could enhance the detection of these metals. The proposed approach offers an efficient way of quantifying PTCs, providing the cement industry with a tool to assess the feasibility of using co-processed waste in cement production. However, further studies should be conducted to optimize the leaching solution and explore its application in various industrial contexts.

## Supplementary information

Below is the link to the electronic supplementary material.

Supplementary file1 (DOCX 3647 KB)

Supplementary file2 (PDF 340 KB)

## Data Availability

The authors declare that the data supporting the findings of this study are available within the article and its supplementary files. Additional information is not publicly available. The research that led to this paper is accessible under restricted conditions at: https://repositorio.ufba.br/xmlui/handle/ri/39254
