# Supplementary material for: Quantification of potentially toxic compounds in anhydrous cements: optimization of leaching by thermal analysis for sustainable production of cements
Source: Environ Sci Pollut Res Int. 2026 May 4;33(16):7626–47. doi: 10.1007/s11356-026-37769-x (PMC13190469; doi:10.1007/s11356-026-37769-x)

**Quantification of Potentially Toxic Compounds in Anhydrous Cements: Optimization of Leaching by Thermal Analysis for Sustainable Production of Cements**

Bruna Souza Rosa^1^ , Samile Raiza Carvalho Matos^2^, Luanne Bastos Barbosa^1^, Ana Paula Kirchheim^3^, Jardel Pereira Gonçalves^1,4^

^1^ Polytechnic School, Federal University of Bahia (UFBA), Salvador, Bahia, Brazil

^2^ Center for Territorial Development (CFDT), Federal University of the South of Bahia, Teixeira de Freitas, Bahia, Brazil.

^3^Institute of Chemistry, Laboratory of Catalysis and Materials (LABCAT), Department of General and Inorganic Chemistry, Federal University of Bahia, Salvador, Bahia, Brazil

^4^Department of Civil Engineering, Federal University of Rio Grande do Sul (UFRGS), Porto Alegre, Rio Grande do Sul, Brazil

^5^Polytechnic School, Interdisciplinary Centre of Energy and Environment (CIENAM), Federal University of Bahia (UFBA), Salvador, Bahia, Brazil

Corresponding author: brunarosa@ufba.br/ https://orcid.org/0009-0005-4848-1995

**Figure S1** Thermal gravimetric analysis of commercial anhydrous cements





**Figure S2**  Thermal gravimetric analysis of anhydrous cements G

**

**

**Figure S3** Schematic LSP Curves of Cationic, Amphoteric, and Oxyanionic Species - Metal solubility


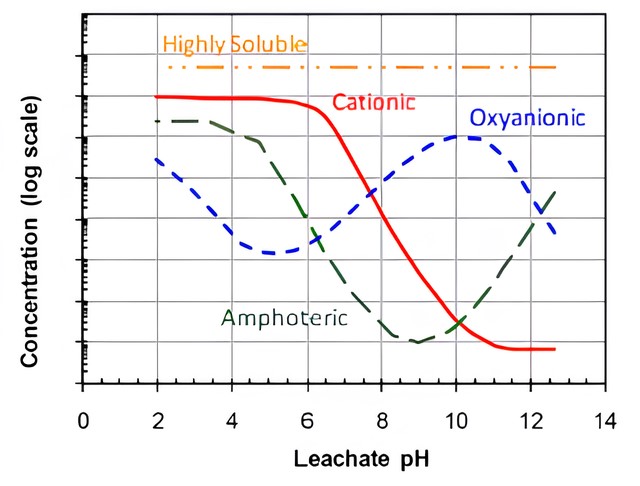


**Ref**: Adaptaded Method 1313 EPA

**Figure S4** Calorimetry of solutions: Tests 2 and 3 of solution for analysis in calorimetry





**Figure S5** The amphoteric species leaching concentrations (µg/L) - Quantitative leaching of oxides amphoteric





**Figure S6** The oxyanionic species leaching concentrations (µg/L) - Quantitative leaching of oxides oxiânions





**Figure S7** The cationic species leaching concentrations (µg/L) - Quantitative leachate of metal cations





**Figure S8** Leaching test procedure according to NBR 10005:2004: (1) stirring the cement in ultrapure water; (2) measuring the pH to define the extracting solution; (3) mechanical shaking in a shaker at 25 °C for 22 ± 2 h; (4) placing the samples in Erlenmeyer flasks, in triplicate; (5) filtering the leachate using a syringe filter; and (6) acidifying an aliquot for metal analysis.


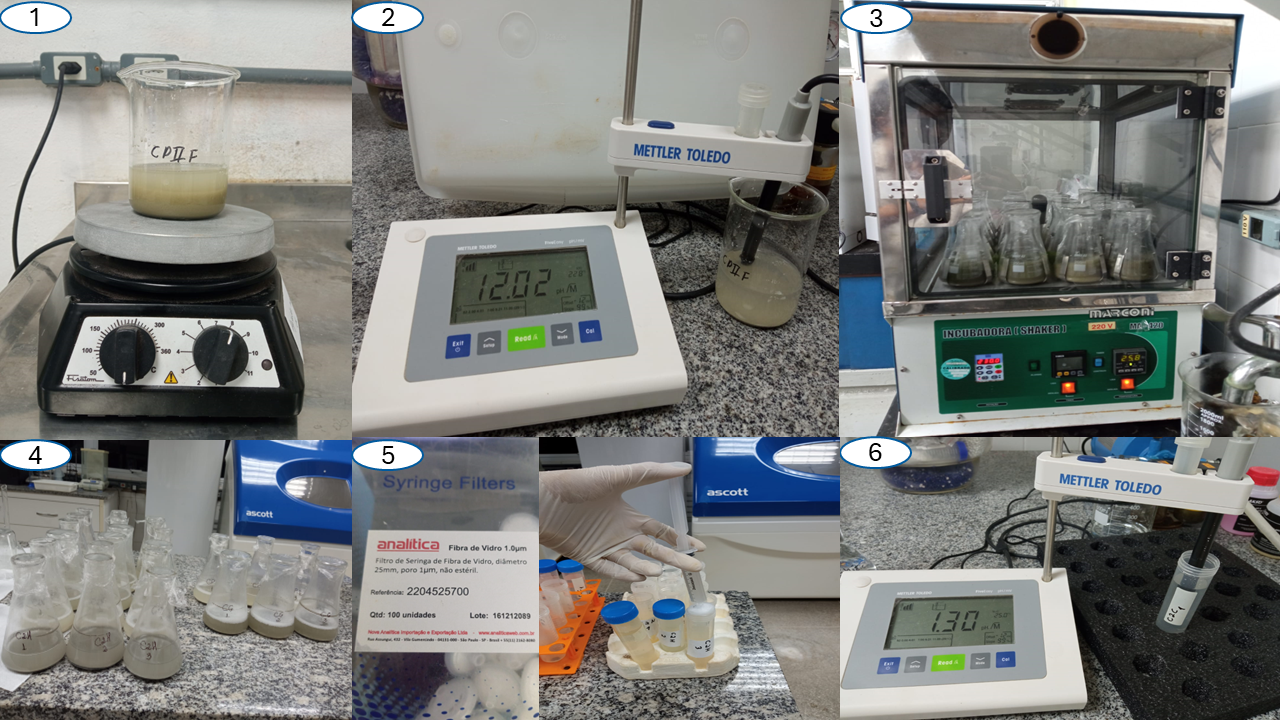


**Figure S9** Steps of the proposed leaching method: (1) preparation of the suspensions in previously decontaminated beakers, with adjustment of the liquid-to-solid ratio (10:1); (2) conditioning of the samples for the test; (3) measurement of the initial pH of the extracting solution; (4) measurement of the pH of the cement–solution mixture; (5) homogenization of the mixture, either by manual stirring (Test 1) or at high rotation speed (Tests 2 and 3); (6) isothermal calorimetry to verify the influence of the extracting solution on the hydration kinetics; (7) obtaining the results and defining the contact time for the leaching test and ICP calibration curve; (8) after filtration using a syringe filter, transfer to Falcon tubes and storage under refrigeration at 4 °C, followed by ICP-OES analysis.


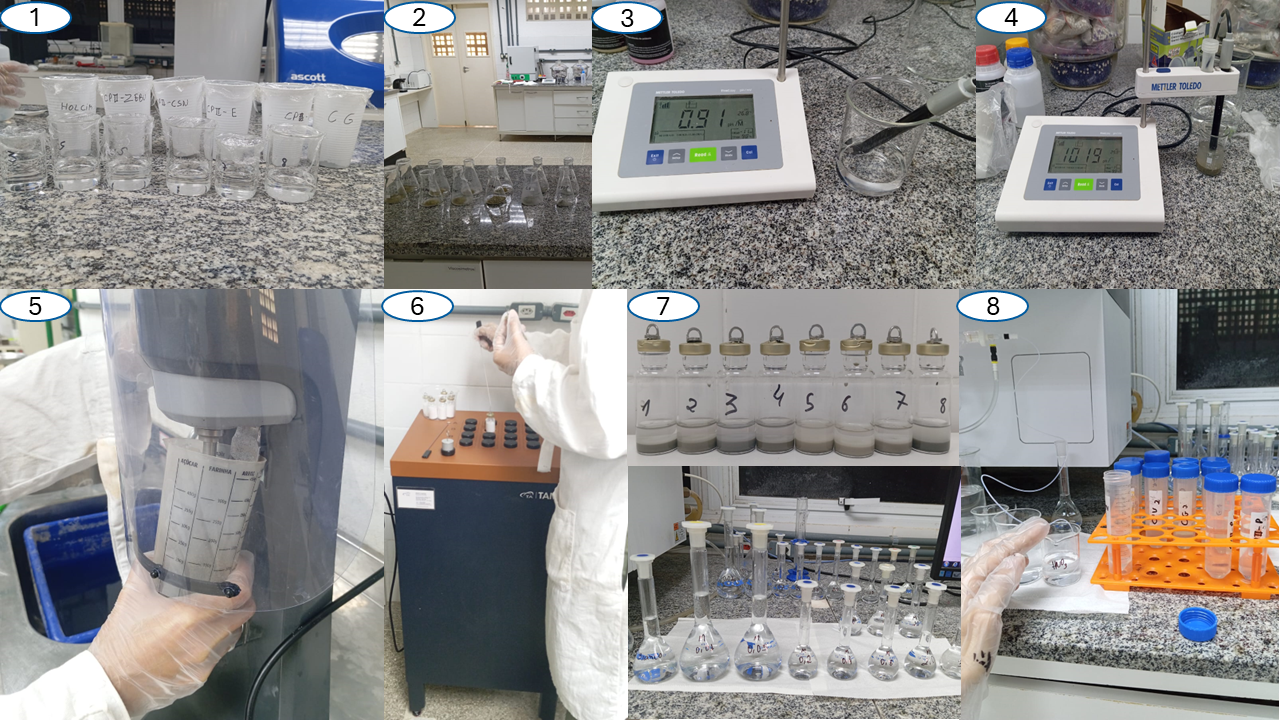

Supplement: Supplementary file 1 — Supplementary file1 (DOCX 3647 KB) [file 11356_2026_37769_MOESM1_ESM.docx]
