# Supplementary material for: Quantification of potentially toxic compounds in anhydrous cements: optimization of leaching by thermal analysis for sustainable production of cements
Source: Environ Sci Pollut Res Int. 2026 May 4;33(16):7626–47. doi: 10.1007/s11356-026-37769-x (PMC13190469; doi:10.1007/s11356-026-37769-x)
Supplement: Supplementary file 2 — Supplementary file2 (PDF 340 KB) [file 11356_2026_37769_MOESM2_ESM.pdf]

Quantification of Potentially Toxic Compounds in Anhydrous Cements: Optimization of Leaching by Thermal Analysis for Sustainable Production of Cements

Bruna Souza Rosa[1] , Samile Raiza Carvalho Matos[2], Luanne Bastos Barbosa[1], Ana Paula Kirchheim[3], Jardel Pereira Gonçalves[1,4]

[1] Polytechnic School, Federal University of Bahia (UFBA), Salvador, Bahia, Brazil
[2] Center for Territorial Development (CFDT), Federal University of the South of Bahia, Teixeira de Freitas, Bahia, Brazil.
[3]Institute of Chemistry, Laboratory of Catalysis and Materials (LABCAT), Department of General and Inorganic Chemistry, Federal University of Bahia, Salvador, Bahia, Brazil
[4]Department of Civil Engineering, Federal University of Rio Grande do Sul (UFRGS), Porto Alegre, Rio Grande do Sul, Brazil
[5]Polytechnic School, Interdisciplinary Centre of Energy and Environment (CIENAM), Federal University of Bahia (UFBA), Salvador, Bahia, Brazil

Corresponding author: brunarosa@ufba.br/ https://orcid.org/0009-0005-4848-1995

Table S1 Limitation of leaching methods

Countries	Method	Aqueous solution	Ratio S/L	Period
Europe	EN 15863	Deionized water	8 mL/cm²	36 days
	EN 15862	Deionized water	12 mL/cm²	24 hours
	UNI EN 12457-1	Deionized water	01:02	24 hours
	UNI EN 12457-2	Deionized water	01:10	24 hours
	UNI EN 12457-3	Deionized water	1:2 e 1:8	6 e 18 hours
	UNI EN 12457-4	Deionized water	01:10	24 hours
	CEN TS 14405	Deionized water	01:10	Calculated based on flow
	NEN 7347	Deionized water	-	64 days
	NEN 7373	Deionized water	1:0,1 e 1:10	Calculated based on flow
	NEN 7375	Deionized water	01:05	64 days
	NF X31-211	Deionized water	01:10	16 a 48 hours
	Method DD CEN/TS 14429:2005	Water with additions of the HNO_3 or NaOH	01:10	4,44,48
United States	Method 1311	Acid solution pH 4,93 ± 0,05 pH 2,88 ± 0,05	01:20	18 hours
	Method 1312	Acid solution pH 4,20 ± 0,05 or pH 5 ± 0,05	01:20	18 hours
	Method 1313	Water with additions of the HNO_3 or NaOH	10 mL/g	24, 48 or 72 hours
	Method 1314	Water or 1 mol $CaCl_2$	10 mL/g	13 days
	Method 1315	Deionized water	9 mL/cm^2	63 days
	Method 1316	Deionized water	10; 5; 2; 1; 0,5 mL/g	24, 48 or 72 hours

Brazil	NBR 10.005	Acid solution pH 4,93 ± 0,05<br>pH 2,88 ± 0,05	01:20	18 hours

Table S2- Codes used in the qualitative analysis of diffractometer data.

XRD	
**Phase**	**ICSD code**
$C_3S.M1$	81100
$C_3S.M3$	94742
C_4AF	161520
$C_2S.\beta$	39006
C_3Ac	1841
CaO	75785
Quartz	62404
Dolomite	10404
Arcanite	79777
Periclase	9863
Mulite	158097
Calcite	28827
Gypsum	151692
Cristobalite	180900
Anidrite III	24473
Hematite	33643
Ettringite	155395
Tobermorite	9005447
Portlandite	15471
Corundum	9770

Table S3 Mass loss temperature ranges in thermogravimetric analysis of Portland cement (PC).

**Anhydrous cement**	
Temperature Range	Phase
100 – 140 °C	$CaSO_4 \cdot 2H_2O$
360 – 470 °C	CH
470 – 800 °C	$CaCO_3$

Table S4 Gypsum mass loss and quantification of portlandite and limestone content by thermogravimetric analysis of cements.

Sample	Gypsum - Loss of mass (%)	Gypsum (%)	CH - Loss of mass (%)	CH (%)	$CaCO_3$ - Loss of mass (%)	$CaCO_3$ (%)	Loss of ignition (LOI)	LOI NBR
CP2-A	0,57	2,72	0,75	3,09	10,17	23,12	12,91	≤12,5
CP2-B	0,46	2,2	0,31	1,27	9	20,45	10,16	≤12,5

CP2-C	0,56	2,68	0,35	1,44	9,86	22,41	11,61	≤12,5
CP2-D	0,87	4,17	0,26	1,07	7,95	18,06	9,38	≤12,5
CP2-E	0,41	1,96	0,41	1,69	9,55	21,71	10,51	≤12,5
CP2-F	0,62	2,96	0,36	1,48	8,42	19,14	9,29	≤12,5
CP V	0,51	2,44	0,52	2,14	3,49	7,94	4,95	≤3,5
CG-A	0,47	2,25	0,11	0,45	0,09	0,2	1,06	≤0,7
CG-B	0,47	2,25	0,0005	0,0019	0,17	0,39	1,49	≤0,7
CG-C	0,9	4,3	0,0005	0,0019	0,21	0,48	1,37	≤0,7
CG-D	0,85	4,06	0,001	0,0041	0,18	0,41	0,84	≤0,7

Table S5 Acidification of samples of each cement

Cement	pH before acidification	pH after acidification
CP2-A	11,5	1,32
CP2-D	11,97	1,03
CP2-E	12,36	0,93
CG-A	12,18	1,42
CP2-B	12,46	1,61
CP2-F	12,36	1,36
CPV	12,36	1,58
CG-D	11,81	1,52
CG-C	11,94	1,46

Table S6 Compatibility between oxides and acids in AES

Chemical composition of anhydrous cement	Compatible acids
CaO	Nitric acid
SiO_2	$NaCO_3$ or KOH
SO_3	Nitric acid / hydrochloric
MgO	Nitric acid
Al_2O_3	Nitric acid
Fe_2O_3	Nitric acid
K_2O	Nitric acid
SrO	Nitric acid
TiO_2	Nitric acid or sulfuric
P_2O_5	$KHPO_4$
Na_2O	-
ZnO	Nitric acid
BaO	Nitric acid or hydrochloric
MnO	Nitric acid
Cl	-
CuO	Nitric acid
V_2O_5	Sulfuric acid or ammonia
ZrO_2	Nitric acid
Cr_2O_3	Nitric acid
NiO	Nitric acid

MoO_3	Ammonia
Ag	Nitric acid
PbO	Nitric acid
Rb_2O	
As_2O_3	Hydrochloric acid
La_2O3	Nitric acid

Table S7 Leaching test 1 (TL.1) under different conditions (before and after leaching for AES analysis)

Cement	pH of the solution	Initial pH	pH after leaching	Acidified pH for reading
CP2-A.1	0,87	6,68	12,17	1,2
C2P-B.1	0,87	7,88	11,95	1,15
C2P-C.1	0,87	7,97	11,73	1,23
C2P-D.1	0,87	7,13	11,81	1,01
C2P-E.1	0,87	7,92	11,92	1,05
C2P-F.1	0,87	7,61	11,9	1,12
CPV.1	0,87	6,32	11,94	1,19
CG-A.1	0,87	10,28	11,86	1,21
CG-B.1	0,87	10,05	11,85	1,18
CG-C.1	0,87	8,51	11,79	1,23
CG-D.1	0,87	8,54	11,85	1,3

Table S8 Leaching test 2 and 3 (TL.2 and TL.3) under different conditions (before and after leaching for AES analysis)

Cement	pH of the solution	Initial pH	pH after leaching	Acidified pH for reading
C5.2	0,91	10,53	11,98	1,31
CP2-D. 2	0,91	10,51	11,63	1,1
CP2-B. 2	0,91	9,77	11,65	1,11
CG-D. 2	0,91	10,25	11,39	1,03
CG-C. 2	0,91	10,21	11,44	1,21
CPV.3	0,91	8,29	10,51	0,89
CP2-D. 3	0,91	7,11	10,17	0,88
CP2-B. 3	0,91	8,15	11,38	0,99
CG-D. 3	0,91	6,4	10,38	0,87

Table S9 – Optimisation and evolution of solutions for leaching tests – Tests 2 and 3

Solution	NaOH (g)	HNO_3 65% (mL)	H_2SO_4 (mL)	Isopropanol (mL)	pH
10	0,1083	1,875	-	-	0,59
11	0,3042	0,5	-	-	1,57
12	0,4092	0,25	-	-	2,16
13	-	1	-	-	0,65
14	0,2917	0,15	-	6	7,35

15	0,2917	0,15	0,05	6	2,2
16	-	-	0,4	-	0,91
17*	-	2,61	-	-	0,68
18*	0,2668	0,25	-	-	2,17

Solution	pH of the solution	pH after mixing (cement* + solution)	pH after 4~5 minutes
10	0,59	10,98	10,68
11	1,57	12,22	12,22
12	2,16	12,12	12,12
13	0,65	9,72	9,72
14	7,35	12,2	12,2
15	2,2	11,59	11,59
16	0,91	8,69	8,68
17*	0,68	10,72	10,72
18*	2,17	12,62	12,62

*cement CP2-F32

Table S10- Hydration parameters - Test 2 and 3 of solution for leaching in AES

**Parameters**	**Sol 2**	**Sol 10**	**Sol 11**	**Sol 12**	**Sol 13**	**Sol 14**	**Sol 15**	**Sol 16**	**Sol 17**	**Sol 18**
Initial setting (h)	40,49	27,17	5,02	4,53	N.I	9,76	13,8	N.I	N.I	5,54
End load (h)	55,16	33,17	7,06	6,66	N.I	14,39	20,94	N.I	N.I	7,26
Duration (h)	14,67	6	2,05	2,14	N.I	4,63	7,14	N.I	N.I	1,73
Duration of induction period-DIP (h)	22,26	20,1	2,09	1,78	N.I	3,64	6,06	N.I	N.I	2,85
Maximum heat flow (mW/g_{cement})	1,18	0,88	4,78	6,79	N.I	1,49	1,44	N.I	N.I	6,02

Table S11 Table of R^2, limit of quantification and limit of detection of the AES utilized

Element	Wavelengths (nm)	R^2	Estimated detection limit (µg/L)	Estimated limit of quantification (µg/L)
Ag	328,068	0,9972	6,05E-04	0,002
Al	394,403	0,9996	0,0476	0,1588
B	249,678	0,9995	5,72E-04	0,0019
Ba	233,527	0,9992	4,02E-05	0,0001
Bi	223,061	0,9996	0,0123	0,0411
Cd	326,106	0,9997	0,0296	0,0988
Co	228,616	0,9993	5,55E-04	0,0019
Cr	205,552	0,9992	6,29E-04	0,0021
Cu	213,598	0,9994	8,40E-05	0,0003
Ga	294,364	0,9994	0,0042	0,014
In	230,606	0,9979	0,0067	0,0223
La	408,672	0,9995	4,71E-04	0,0016
Li	323,261	0,9995	0,057	0,19
Mn	260,569	0,9981	1,74E-04	0,0006
Ni	341,476	0,9991	0,0034	0,0112
Pb	405,783	0,9993	0,0048	0,016
Sb	206,833	0,9993	0,0034	0,0114
Sr	216,596	0,9991	0,0068	0,0227
Tl	276,787	0,9994	0,0115	0,0383
V	292,402	0,9994	3,89E-04	0,0013
Zn	206,2	0,9991	0,0016	0,0052
